# Type III Methyltransferase M.NgoAX from *Neisseria gonorrhoeae* FA1090 Regulates Biofilm Formation and Interactions with Human Cells

**DOI:** 10.3389/fmicb.2015.01426

**Published:** 2015-12-21

**Authors:** Agnieszka Kwiatek, Agnieszka Mrozek, Pawel Bacal, Andrzej Piekarowicz, Monika Adamczyk-Popławska

**Affiliations:** ^1^Institute of Microbiology, Faculty of Biology, University of WarsawWarsaw, Poland; ^2^Laboratory of Theory and Applications of Electrodes, Faculty of Chemistry, University of WarsawWarsaw, Poland

**Keywords:** *Neisseria gonorrhoeae*, restriction modification, NgoAX, adhesion and invasion of epithelial cells, biofilm, phase variation

## Abstract

*Neisseria gonorrhoeae* is the etiological factor of the sexually transmitted gonorrhea disease that may lead, under specific conditions, to systemic infections. The gonococcal genome encodes many restriction modification (RM) systems, which main biological role is to defend the pathogen from potentially harmful foreign DNA. However, RM systems seem also to be involved in several other functions. In this study, we examined the effect of inactivation the *N. gonorrhoeae* FA1090 *ngoAXmod* gene encoding M.NgoAX methyltransferase on the global gene expression, biofilm formation, interactions with human epithelial host cells and overall bacterial growth. Expression microarrays showed at least a twofold deregulation of a total of 121 genes in the NgoAX knock-out mutant compared to the wild-type (wt) strain under standard grow conditions. Genes with changed expression levels encoded mostly proteins involved in cell metabolism, DNA replication and repair or regulating cellular processes and signaling (such as cell wall/envelop biogenesis). As determined by the assay with crystal violet, the NgoAX knock-out strain formed a slightly larger biofilm biomass per cell than the wt strain. Live biofilm observations showed that the biofilm formed by the gonococcal *ngoAXmod* gene mutant is more relaxed, dispersed and thicker than the one formed by the wt strain. This more relaxed feature of the biofilm, in respect to adhesion and bacterial interactions, can be involved in pathogenesis. Moreover, the overall adhesion of mutant bacterial cells to human cells was lower than adhesion of the wt gonococci [adhesion index = 0.672 (±0.2) and 2.15 (±1.53), respectively]; yet, a higher number of mutant than wt bacteria were found inside the Hec-1-B epithelial cells [invasion index = 3.38 (±0.93) × 10^5^ for mutant and 4.67 (±3.09) × 10^4^ for the wt strain]. These results indicate that NgoAX knock-out cells have lower ability to attach to human cells, but more easily penetrate inside the host cells. All these data suggest that the NgoAX methyltransferase, may be implicated in *N. gonorrhoeae* pathogenicity, involving regulation of biofilm formation, adhesion to host cells and epithelial cell invasion.

## Introduction

Gonorrhea, caused by *Neisseria gonorrhoeae*, is the second most common sexually transmitted disease in the world. Every year, this exclusively human pathogen infects about 106 million people (WHO, 2012). The disease is associated with low mortality but high prevalence, has serious socio-economic effects and is a public health problem worldwide. *N. gonorrhoeae* can infect the urogenital tract, anus, or throat. Gonococcal infections in men principally cause inflammation of the urethra, which can lead to such complications as urethral strictures, inflammation of the epididymis or the prostate gland (Edwards and Apicella, 2004; Marrazzo et al., 2010; Ison, 2011). The mucous membrane of the cervix is the most common site of infection of *N. gonorrhoeae* in women (Ison, 2011), in which, gonococci may cause asymptomatic or symptomatic cervical infections, or upper genital tract disease (Bozicevic et al., 2006; Edwards and Butler, 2011). Undetected or untreated gonorrhea can lead to very serious complications. These include: pelvic inflammatory disease, ectopic pregnancy, infertility in women and men, and systemic infections (Holmes, 1999; Ison, 2011; Unemo and Nicholas, 2012). In addition, gonorrhea may increase the risk of human immunodeficiency virus (HIV) transmission as suggested by many studies (for review see Jarvis and Chang, 2012). A recent study demonstrate that *N. gonorrhoeae* libarates a carbohydrate, heptose-monophosphate, that elicits an innate immune response and drives HIV-1 expression (Malott et al., 2013). Phase variation is a well-characterized mechanism by which numerous bacterial species, including *N. gonorrhoeae*, introduce phenotypic diversity within a population and increase pathogenicity (Snyder et al., 2001; Jordan et al., 2005; Srikhanta et al., 2009).

The main biological role of bacterial RM systems is to defend against the attack of potentially harmful foreign DNA. Yet, RM systems seem also to exhibit several other functions (for review see Vasu and Nagaraja, 2013). The role of RM systems in epigenetic signaling has been investigated by several research groups (Fox et al., 2007a; Srikhanta et al., 2009, 2010, 2011). RM systems have been shown to be involved in epigenetic regulation, which suggests their involvement in adaptive evolution by regulation of the global gene expression pattern (Low et al., 2001; Srikhanta et al., 2010). Type III RM system were also described affecting the interactions of pathogenic bacteria with host cells (Srikhanta et al., 2009). Through the modulation of genetic variation, RM may also affect the evolution rate (Arber, 2000). It seems that RM plays an important role in co-evolutionary interactions between mobile genetic elements and their hosts (Sneppen et al., 2015). RM systems are compared to toxin–antitoxin systems (Mruk and Kobayashi, 2014). It has been also shown that restriction products can stimulate their homologous recombination with the host genome (Price and Bickle, 1986; Arber, 2000). RM systems behave like selfish genetic elements that promote their own survival and increase their own turnout (Kobayashi, 2001; Vasu and Nagaraja, 2013). In addition, RM systems control the immigration of foreign DNA by restriction endonuclease function. This barrier allows controlling speciation of bacteria, which is associated with genetic isolation (Jeltsch, 2003).

Bacteria use DNA adenine methylation (Dam) as an epigenetic signal by which additional information is imparted to the DNA. Dam methyltransferases play, among others, a pivotal role in regulation of gene expression and phase variation (Marinus and Casadesus, 2009). However, as we previously demonstrated, *N. gonorrhoeae* FA1090 does not encode a Dam methyltransferase and biological functions of Dam are suggested to be taken over by another system (Kwiatek et al., 2014).

Restriction modification systems are ubiquitous in bacteria and include two enzymatic activities: endonucleolytic and methylating. Restriction endonucleases and methyltransferases recognize the same specific nucleotide sequence in double-stranded DNA, but differ in catalyzed reaction. Most endonucleases cut the foreign double-stranded DNA, when it is not specifically methylated, by hydrolysis of phosphodiester bonds. In contrast, methyltransferases modify nucleotides present in specific sequences of the host DNA by attaching to them a methyl group. Such modifications protect the host DNA from endonucleolytic restriction (Fox et al., 2007b; Rao et al., 2014).

Restriction and modification systems have been divided into four types: I, II, III, and IV, according to their mode of action and distribution of restriction, modification, and specificity functions within the enzyme subunits (Roberts et al., 2015).

Type III restriction-modification systems are the most poorly characterized among all types of RM systems (Williams, 2003; Rao et al., 2014). Rebase, the library of RM systems, revealed more than 155 confirmed enzymes belonging to type III RM (Roberts et al., 2015). Type III RM systems consist of two closely spaced genes, *mod* and *res* (Hümbelin et al., 1988). *Mod* genes encode the methyltransferase subunit Mod, responsible for sequence recognition and modification, while *res* genes encode the restriction endonuclease subunit. Methyltransferases are responsible for recognizing the asymmetric target sequence and methylation reaction catalysis. Enzymes of this type operate independently of the restriction endonuclease (Rao et al., 2014). Type III methyltransferases methylate adenine only on one strand of the recognized DNA sequence and convert it into an N6-methyladenine (Dryden et al., 2001). Restriction activity is performed only by a complex formed of Mod and Res subunit. Res subunit alone has no activity.

Few recent publications suggest that DNA methyltransferases belonging to type III RM systems play a role of epigenetic mechanisms, which control gene expression. Control of multiple gene expression by type III RM systems has been described in several human pathogenic bacteria (Srikhanta et al., 2005, 2009, 2011; Bayliss et al., 2006). Moreover, in *Haemophilus influenzae* and some other bacteria type I and III DNA methyltransferases undergo phase variation, which increases their potential role in controlling gene expression (Ryan and Lo, 1999; De Bolle et al., 2000; Srikhanta et al., 2005; Fox et al., 2007a; Broadbent et al., 2010; Seib et al., 2015).

Sixteen potential RM systems, in the chromosome of the *N. gonorrhoeae* FA1090 strain (accession no. AE004969), have been identified using bioinformatic analysis (Stein et al., 1995). However, only a few of them have been experimentally studied (Rebase). Among *N. gonorrhoeae* FA1090 RM systems, only two belong to the type III group (Stein et al., 1995). The first one is NgoAXII (encoded by the *ngo0641* gene, also called *modA13*), for which the recognition sequence has been determined to be 5′ AGAAA 3′. Expression of the NgoAXII methyltransferase undergoes phase variation associated with the control of expression of many genes reassembled in a *phasevarion* (Srikhanta et al., 2009). The second type III RM system in *N. gonorrhoeae* FA1090 is NgoAX, encoded by two genes *ngoAXmod* (or *ngo0545*, also called *modB1*, Srikhanta et al., 2009) and *ngoAXres* (*ngo0546)*, which respective products are a methyltransferase and restrictase. Recognition sequence of the NgoAX methyltransferase is: 5′-CCACC-3′ as was experimentally demonstrated. Like other type III methyltransferases, it methylates only one strand of the dsDNA (Adamczyk-Poplawska et al., 2009). We have previously observed that the restriction phenotype associated with NgoAX switches randomly when cloned in *Escherichia coli*. This phase variation was determined to be related to a variability in the number of 5′-CCAAC/G-3′, pentanucleotide repeats present at the 5′-end of the *ngoAXmod* coding region, which causes a frameshift of the *ngoAXmod* gene (Adamczyk-Poplawska et al., 2009). Such change resulted in switching the M.NgoAX methyltransferase (as well as the whole RM system) from ON to OFF state.

In this work we studied the phenotypic effect of *ngoAXmod* inactivation in *N. gonorrhoeae* FA1090. The influence of the lack of DNA methylation by NgoAX methyltransferase on global gene expression was also evaluated. Moreover, inactivation of NgoAX on biofilm formation, interactions with human epithelial host cells and overall bacterial growth were examined.

## Materials and Methods

### Bacterial Strains Growth Conditions

*Escherichia coli* strain Top10 [F’[lacI^q^ Tn10(Tet^r^)] *mcrA* Δ(*mrr*-*hsdRMS*-*mcrBC*) *φ80lacZΔM15 ΔlacX74 nupG recA1 araD139* Δ(*ara*-*leu*)*7697 galE15 galK16 rpsL*(Str^r^) *endA1* AAA^-^] was grown in Luria-Bertani (LB) broth (Difco) or on LB agar plates at 37°C. When needed, medium was supplemented with kanamycin (30 μg/ml) or chloramphenicol (34 μg/ml) or ampicillin (100 μg/ml).

*Neisseria gonorrhoeae* strains were grown on GCB agar base (Difco) supplemented with 1% *Kellogg’s* supplement and 1% hemoglobin at 37°C in 5% CO_2_ or in GCB broth with 1% *Kellogg’s* supplement and 0.043% NaHCO_3_, according to Dillard (2006). The following antibiotics were used, when needed: kanamycin (30 μg/ml) or chloramphenicol (0.75 μg/ml).

### Construction of *N. gonorrhoeae* Mutant Strains: The Knock-Out Mutant in NgoAX RM System (*ngoAXmod::km*) and the Complementation Mutant (*igatrpb::ngoAXmod*)

To construct the NgoAX knock-out mutant, we replaced the wt *ngoAXmod* gene on the FA1090 chromosome with a disrupted by antibiotic cassette allele.

The pNgoAXP plasmid encoding the NgoAX restriction-modification system from *N. gonorrhoeae* FA1090 (Adamczyk-Poplawska et al., 2009) was digested with MunI. In effect a deletion of a 600-bp fragment within the *ngoAXmod* gene encoding the Mod_NgoAX_ subunit of NgoAX was generated. The obtained fragment was ligated with the EcoRI fragment from the pDIY-km plasmid (Dziewit et al., 2011), containing a *km* cassette active in *N. gonorrhoeae* (Kwiatek et al., 2014). The resulting plasmid was linearized with MluI and used to transform piliated, i.e., competent *N. gonorrhoeae* cells. Linearization of the vector forced the replacement of the wt *ngoAXmod* gene by the *ngoAXmod::km* allele, by the occurrence of double crossing-over. *N. gonorrhoeae* FA1090 colonies lacking the NgoAX RM system (NgoAX knock-out mutants) were selected on kanamycin containing GCB plates.

To construct the *N. gonorrhoeae* complementation mutant, the *ngoAXmod* gene was amplified from chromosomal DNA of *N. gonorrhoeae* FA1090 (Gene Bank: AE004969, ATCC 700825) by PCR with Smamod and Nhemod primers (see Supplementary Table S1 for primer sequences), using PfuUltra II Fusion HS DNA polymerase (Agilent Technologies), according to the manufacturer’s protocol. The resulting fragment of 2094 bp was ligated with the PCR fragment (6578 bp) obtained by amplification using the pMPMigatrpBopaCM vector (pMPMA4Ω vector with cloned intergenic region *iga-trpb* and *cm* cassette) as template and primers Smatrpb and Nheiga (see Supplementary Table S1 for primer sequences). In the obtained construct, the *ngoAXmod* gene is under control of a strong constitutive promoter as previously described (Ramsey et al., 2012). The recombinant vector was linearized with EcoRV and used to transform the *N. gonorrhoeae ngoAXmod::km* strain. *N. gonorrhoeae* cells, in which the interrupted *ngoAXmod* gene was complemented with the wt *ngoAXmod* gene, inserted between *iga* and *trpb* genes, were selected on GCB plates containing 0.75 μg/ml chloramphenicol and 30 μg/ml kanamycin. The resulting recombinant strain was called *N. gonorrhoeae igatrpb::ngoAXmod* (complementation mutant).

Disruption of the *ngoAXmod* gene by the *km* cassette and introduction of the wt *ngoAXmod* gene into the region between *iga* and *trpb* genes in *N. gonorrhoeae* FA1090 was verified by PCR, sequencing and Southern Blot. For Southern Blot, isolated, from the wt *N. gonorrhoeae*, the knock-out and complementation mutants, 1.0 μg of chromosomal DNAs were digested with MluI restriction enzyme. Obtained fragments were separated on agarose gels (0.7%) at 130 V for 2 h and Southern alkali transfer, followed by hybridization were performed as described by Sambrook and Russell. We used non-radioactively labeled *km* or chloramphenicol cassette (DIG-High Prime, Roche) as probe (data not shown).

### Field Emission Scanning Electron Microscopy (FE SEM)

For FE SEM, *N. gonorrhoeae ngoAXmod::km*, *igatrpb::ngoAXmod* and wt strains were harvested from 24 h GCB plates and suspended in GCB broth. OD_600_ were adjusted to 0.05, corresponding to 4 × 10^7^ cfu/ml. Bacteria were then cultivated on cover glasses, placed in Petri dishes in GCB broth at 37°C in 5% CO_2_. After 24 h, samples for FE SEM were prepared as previously described (Kwiatek et al., 2014) and observed with MERLIN Carl Zeiss FE SEM Microscope.

### Live Cell Confocal Microscopy (SCLM)

We quantified live biofilm development after 16 h growth on Glass Bottom Microwell Dishes (MatTek Corporation) as previously described by Kwiatek et al. (2014), using a Nikon Eclipse Ti (A1) microscope and the *NIS-ELEMENTS* interactive software.

### Microtiter-Plate Adherence Assay

Microtiter-plate adherence assay was performed as described previously (Stepanovic et al., 2000; Kwiatek et al., 2014). Briefly, *N. gonorrhoeae* wt strains were harvested from 24 h GCB agar and OD_600_ = 0.05 suspensions were made in GCB broth supplemented with *Kellogg’s* supplement and MgSO_4._ (5 mM). Bacteria were then plated into 96-well microtiter plates and cultivated for 24 h at 37°C in 5% CO_2_. After the OD_600_ measurement, the supernatant was aspirated, and each well was extensively washed with PBS in order to remove all non-adherent bacteria. The attached bacteria were processed as previously described and stained with crystal violet (0.8%). After extensive washing and resolubilising the dye bound to biofilm-forming cells, OD_570_ was measured. Experiments were carried out three times and results were averaged. The significance of results was determined by Student’s *t*-test (*P* < 0.05).

### RNA Isolation

RNA isolation was performed as described previously (Kwiatek et al., 2014). Total RNA was isolated from cells growing on one GCB agar plate (no pooled samples) using the High Pure RNA Isolation Kit (Roche) and DNA-*free^TM^*, DNase Treatment and Removal (Ambion), according to the manufacturer’s recommendations. From one plate, approximately 20–30 μg of total bacterial RNA was purified. RNA quality was evaluated using and 2100 Bioanalyzer (Agilent Technologies) and NanoDrop 2000 (Thermo Scientific).

### Microarray Experiments

Two color DNA microarray analysis was performed using the Agilent-034141 array as described in details by Kwiatek et al. (2014). The Cy5 dye was used to label the cRNA *ngoAXmod::km* strain and the Cy3 dye to label the cRNA *N. gonorrhoeae* FA1090 strain. Four wt and four mutant biological replicates were studied.

### Microarray Analysis

Data files from the Agilent G2565CA Microarray Scanner System were loaded into the GeneSpring (Agilent Technologies, version 12.5) and analyzed as described previously (Kwiatek et al., 2014). Variations were presented as the ratio of gene expression of wt gonococcal strain over expression of the same gene for the NgoAXP knock-out mutant. Genes with divergent expression of ≥1.5 or 2-fold and a *P*-value < 0.05 were chosen. Microarray data have been deposited in the NCBI’s and are accessible through Gene Expression Omnibus series accession number GSE71703^1^ (Edgar et al., 2002).

### Real-Time qRT-PCR

Ten micro gram of total RNA (used for microarrays) were submitted to reverse transcription using the Maxima First Strand cDNA Synthesis Kit (Thermo Scientific). Real-time PCR (5 × HOT FIREPol^®^ EvaGreen^®^ qPCR Mix Plus, Solis BioDyne) was carried out on The Applied Biosystems^®^ StepOne^TM^ Real-Time PCR Systems (Life Technologies). For each gene of interest we used HPLC-purified oligonucleotide primers (see Supplementary Table S1).

The comparative threshold cycle (Ct) method was used to relative quantification of gene transcription. The 16S rRNA gene was used as an internal reference to normalize the relative amount of target cDNA. Data represent averages of four independent samples.

### Gonococcal Cell Viability Assay

*Neisseria gonorrhoeae* cells were harvested from GCB agar and cell suspensions were made in GCB broth with *Kellogg*’s supplement, 5 mM MgSO_4,_ and 0.043% Na_2_CO_3_ (10^7^ cells/ml). Bacterial cultures were incubated at 37°C with gentle shaking for 8 h. Every hour, samples were taken and the number of viable cells was determined using the BacTiter-Glo Microbial Cell Viability Assay (Promega) as previously described (Kwiatek et al., 2014). As negative control we used GCB broth with supplements. Signal detection was performed using the luminometer Glomax multiplus (Promega). All measurements were carried out three times and results were averaged. The significance of results was determined by Student’s *t*-test (*P* < 0.05).

### Hec1-B Cell Culturing

The human adenocarcinoma endometrial cell line Hec-1-B, acquired from the ATCC (ATCC HTB113), was cultivated in DMEM (Sigma–Aldrich) supplemented with 10% fetal bovine serum (Cytogen), 2 mM glutamine (Cytogen) and 1 mM sodium pyruvate (Sigma–Aldrich). Cells were incubated at 37°C in 5% CO_2_ and passaged every 5 days.

### Infection of Hec-1-B Cells with *N. gonorrhoeae*

Bacteria were plated on GCB agar plates 2 days before infection and grown at 37°C, in a 5% CO_2_-containing atmosphere. Then, colonies were checked under a light microscope for pilus and Opa expression status by colony morphology (Swanson, 1978). Appropriate clones were picked and streaked onto new GCB agar plates. The plates were then grown for no more than 16 h to avoid autolysis of bacteria. Bacteria were scraped from GCB agar plates in GCB broth and used to infect the epithelial cells at a multiplicity of infection of 100.

### Adhesion Assay of *N. gonorrhoeae* to Human Cells

Human Hec-1-B cells were seeded in 24-well plates (Cellstar) at a density of 10^5^ cells/well. After incubation for 5 days at 37°C in 5% CO_2_, bacteria were added. Four hour post infection, the medium was discarded from one set of wells, and human cells were washed extensively with pre-warmed PBS and then lysed with 0.5% saponin (Sigma–Aldrich). Released bacterial cells were plated for enumeration of bacteria associated to human cells: human cell-associated colony forming units (CFU). At the same time, the supernatant was collected from a parallel set of infected cultures and bacteria present in this supernatant were plated on GCB agar for enumeration of bacteria not associated to human cells. Hec-1-B cells from this parallel set of wells were also lysed and released bacteria plated on GCB agar for CFU enumeration. The total CFU was defined as the sum of CFU of bacteria not associated to human cells and bacteria released from human cells. The adhesion index was the quotient of dividing the cell-associated CFU by the total CFU (Hopper et al., 2000; Kwiatek et al., 2014). Tests were carried out at least three times and results were averaged. The significance of results was determined by the Student’s *t*-test (*P* < 0.05).

### Invasion Assay of *N. gonorrhoeae* into Human Cells

Hec-1-B cells were seeded and infected as described above for adhesion assays. Invasion assays were performed essentially as described by Hopper et al. (2000). Four hour after infection, sets of wells were washed with pre-warmed PBS and further incubated for 60 min with DMEM containing 200 μg/ml of gentamicin (Gibco) in order to kill extracellular bacteria. Subsequently, cultures were washed extensively again to remove gentamicin and then lysed as described above. Released bacteria were plated for enumeration of intracellular CFU. At the same time point, separate sets of cultures were processed for calculation of adhesion index. The invasion index was calculated by dividing the number of intracellular bacteria by the adhesion index. All infections were performed in triplicate. The significance of results was determined by the Student’s *t*-test (*P* < 0.05).

### Enzymes, Oligonucleotides and Chemicals

Enzymes were acquired from Thermo Scientific. Kits for chromosomal DNA purification, DNA clean-up and plasmid DNA extraction were obtained from A&A Biotechnology, Gdansk, Poland. Unless otherwise noted, other chemicals were acquired from Sigma–Aldrich. Primers for DNA amplification were purchased from the Institute of Biochemistry and Biophysics (Poland) or from Sigma–Aldrich (HPLC-purified primers for qRT-PCR).

### Other Methods

Routine methods were carried out as described by Sambrook and Russell (2001).

### Computer Analysis

DNA and protein sequences were analyzed with BLAST, Genebank, KEGG and Uniprot databases. COG were assigned using http://www.ncbi.nlm.nih.gov/Structure/cdd/wrpsb.cgi. Predicted outer membrane proteins were checked using http://db.psort.org/.

## Results

### Expression of Type III Methyltransferases in *N. gonorrhoeae* FA1090

According to Rebase (Roberts et al., 2015) and literature data (Stein et al., 1995; Srikhanta et al., 2009), *N. gonorrhoeae* FA1090 encodes two type III RM systems: NgoAX, encoded by the *ngo0545* gene, also called *ngoAXmod* (encoding the Mod subunit) and *ngo0546* (coding the Res subunit) and the NgoAXII system, encoded by the *ngo0641* gene, called *ngoAXIImod* (Mod) and *ngo0640* (Res). By qRT-PCR, we determined the relative level of expression of genes encoding methyltransferases in comparison to the level of expression of a housekeeping gene, encoding 16S rRNA. Our results indicated that the *ngoAXmod* gene is expressed at 4.488 (±1.11) copies/million 16S rRNA copies and *ngoAXIImod* at 0.621 (±0.067) copies/million.

### Construction of the *N. gonorrhoeae* NgoAX Knock-Out Mutant and Complementation Strain

To study gene expression and selected physiological aspects of neisserial pathogenesis in the presence or absence of NgoAX methyltransferase activity, two gonococcal mutants were obtained. Mutant strains were constructed using suicide plasmids, by the gene replacement method. A plasmid with the *ngoAXmod* gene with cloned *km* cassette was used for interruption of the chromosomal *ngoAXmod* gene, resulting in the *N. gonorrhoeae ngoAXmod::km* strain (otherwise called the NgoAX knock-out strain).

To obtain the *N. gonorrhoeae* complementation mutant, we cloned the wt *ngoAXmod* gene into the intergenic region between *iga* and *trpb* locus present on the pMPMigatrpBopaCM plasmid. In this vector, the cloned *ngoAXmod* gene was under control of a constitutive promoter as described previously and a chloramphenicol cassette was used for recombinant vector selection (Ramsey et al., 2012). This plasmid was transformed into the *N. gonorrhoeae ngoAXmod::km* strain, generating *N. gonorrhoeae igatrpb:: ngoAXmod*. The obtained *N. gonorrhoeae* complementation strain was both, kanamycin and chloramphenicol resistant: the *km* cassette disrupted the original *ngoAXmod* locus and the wt *ngoAXmod* copy was inserted into the chromosome, followed by the chloramphenicol cassette for recombinant cell selection.

To confirm that the constructed mutant strains contain only the desired insertions PCR, gene sequencing and Southern Blot were performed. With Smamod and Nhemod primers (Supplementary Table S1), we performed PCR on genomic DNA isolated from the wt and mutants strains. As expected, a PCR product of 2095 bp for wt gene or a 2552-bp long PCR for interrupted *ngoAXmod:: km* were obtained, while both products were generated in case of the *igatrpb::ngoAXmod* mutant. With AXPleft and AXPright primers (Supplementary Table S1), we obtained a PCR product of 1435 bp for the wt strain, 1893-bp DNA fragment for the knock-out mutant in the NgoAX RM system, and both products for the complementation mutant. PCR analysis performed with Trpb and Iga primers (Supplementary Table S1) on genomic DNA isolated from the wt strain and complementation mutant resulted, as expected, in a 1134-bp fragment for the wt strain or a 4286-bp long PCR for the *igatrpb::ngoAXmod* mutant, corresponding to the intergenic region and inserted *ngoAXmod* gene (data not shown).

### Absence of the M.NgoAX Methyltransferase Enhances *N. gonorrhoeae* Growth

*Neisseria gonorrhoeae* cells were grown in liquid medium with gentle agitation at 37°C. To measure the viability of *ngoAXmod::km* mutant and control gonococcal strains, we measure a luminescent signal, proportional to the number of viable cells by using the Microbial Cell Viability Assay (BacTiter-Glo^TM^).

**FIGURE 1:**
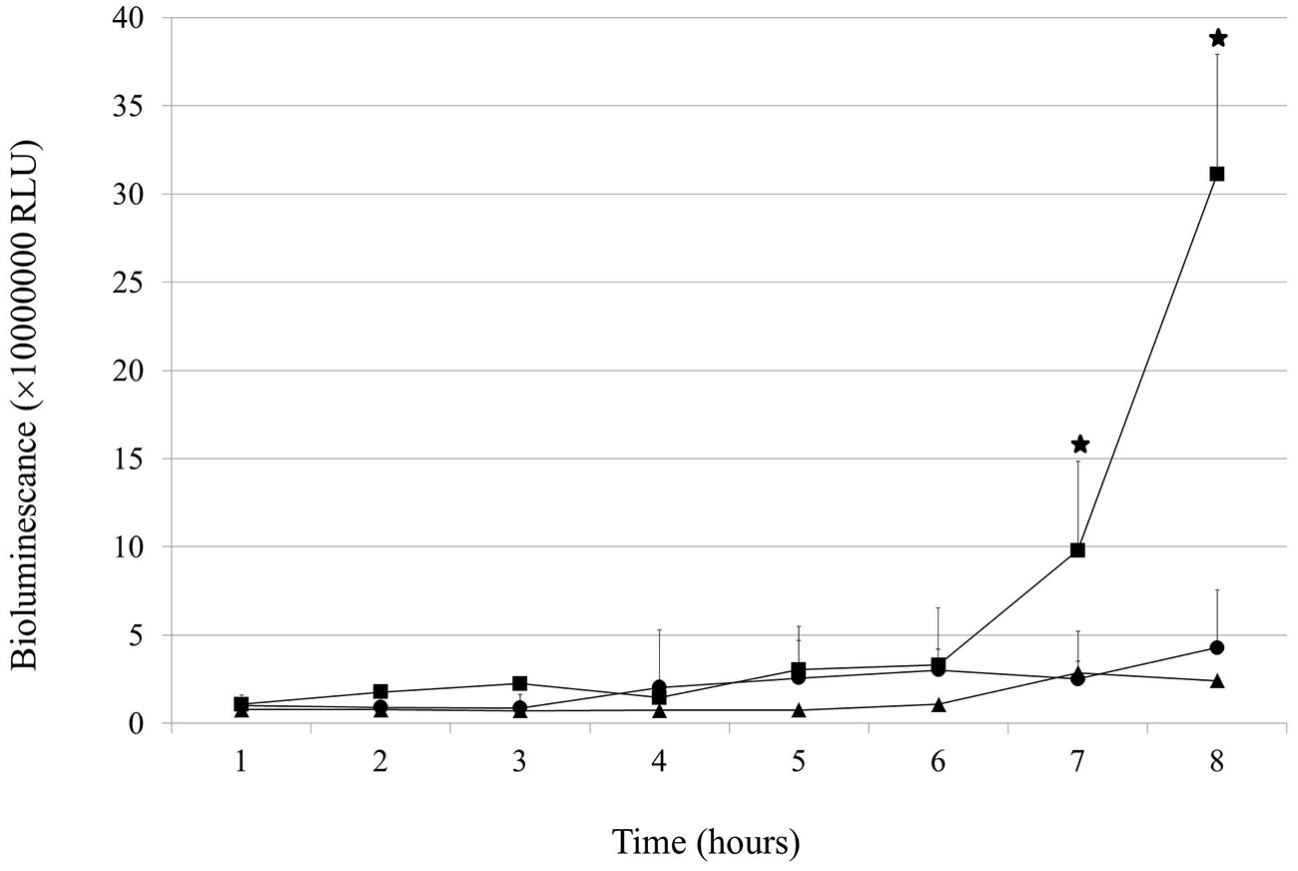
**Effect of *ngoAXmod* disruption on *Neisseria gonorrhoeae* growth.** Growth and viability of the wt *N. gonorrhoeae* (triangle), *ngoAXmod::km* mutant (square) and *igatrpb::ngoAXmod* mutant (circle) were measured for 8 h using the BacTiter-Glo^TM^ Microbial Cell Viability Assay kit. The amount of viable cells was directly proportional to the produced bioluminescence. The asterisk represents the statistical difference in comparison to the wt strain (*P* < 0.05).

Under the applied conditions, the growth of the wt gonococcal cells was very slow and a plateau-like phase was achieved after approximately 5 h (**Figure 1**). After 8 h of growth, wt cells reached the level of 4.28 (±3.3) × 10^6^ RLU (relative light units). In contrast, NgoAX knock-out cells kept growing exponentially during 5 h and after 7 and 8 h reached the level of 9.79 (±5.05) and 31.1 (±6.79) × 10^6^ RLU, respectively (which is statistically different from the level of the wt cells: *P* < 0.05 at both laps times). Complementation of the *ngoAXmod* gene mutation (*igatrpb::ngoAXmod* mutant) restored the growth to a similar level as for the wt gonococci (*P* > 0.05).

### Impact of the *ngoAXmod* Gene Inactivation on Gonococcal Gene Expression

In the NgoAX knock-out mutant, a total of 121 genes were deregulated (38 down and 83 up) at least twofold as compared to the wt strain under standard growth conditions. This group of genes represented 5.61% of the total gene pool. When the cut-off was positioned at 1.5 fold, the expression of 249 genes (11.54% of total genes) was changed in the *ngoAXmod::km* mutant: 106 genes were downregulated and 143 upregulated (Supplementary Table S2). Expression of randomly chosen genes was examined by qRT-PCR (Supplementary Table S2).

**FIGURE 2:**
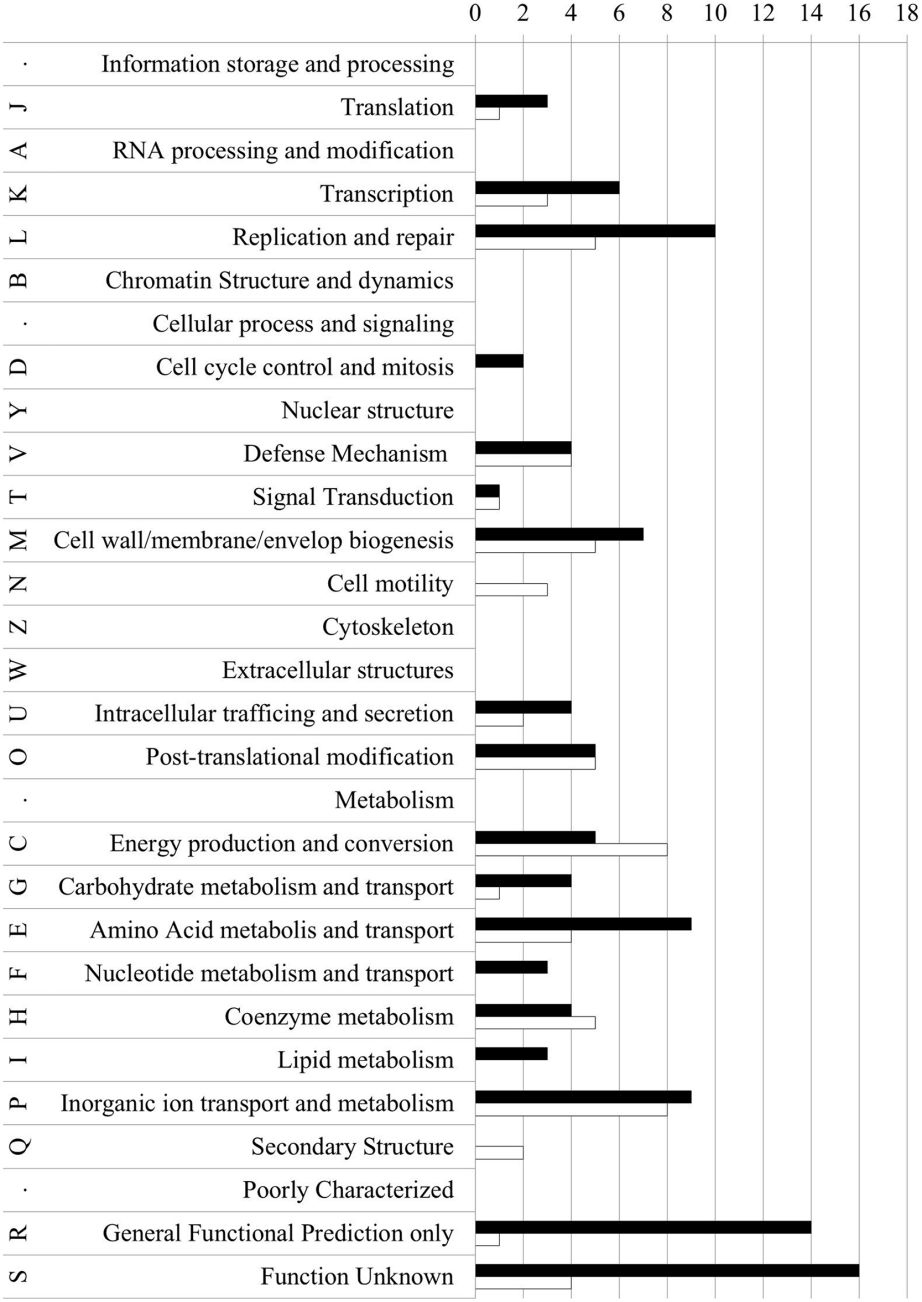
**Clusters of orthologous groups classification of proteins encoded by up- and down-regulated genes in the *NgoAX* knock-out *N. gonorrhoeae* versus the wt strain.** Letters represent COGs categories; numbers – the number of proteins in each category; black bars – proteins encoded by up-regulated genes; white bars – proteins encoded by down-regulated genes.

For each gene with deregulated expression, we determined the COG category (cluster of the orthologous gene, Natale et al., 2000; Tatusov et al., 2000) category (Supplementary Table S2; **Figure 2**). Most genes with affected expression encoded hypothetical proteins with unknown function (S or R COG category: 35 gene products) or proteins in which no conserved domains (87 gene products). Sixty five genes with changed expression levels encoded proteins involved in cell metabolism (mostly categories C and P). Fifteen genes encoded proteins involved in DNA replication and repair (category L). Forty three genes encoded proteins regulating cellular processes and signaling, among them 12 genes encoded proteins involved in cell wall/envelop biogenesis (M category) and 10 – proteins engaged in posttranslational modifications (O category).

Among the affected genes, encoding proteins with unknown functional COG, many were involved in biofilm formation and/or adhesion. For example, the operon containing *ngo0095*–*ngo0098* genes, involved in pilus formation, was downregulated. Other downregulated genes involved in adhesion were those encoding adhesins MafA and MafB (*ngo1393* and *ngo41068*) or OpaD, encoded by *ngo1513*. In contrast, expression of the *ngo0180* gene, encoding a Maf-like protein, was upregulated.

Detailed information about deregulated genes can be found in Supplementary Table S2.

### Biofilm Formation by *N. gonorrhoeae* FA1090 is Modulated by M.NgoAX Methyltransferases

**FIGURE 3:**
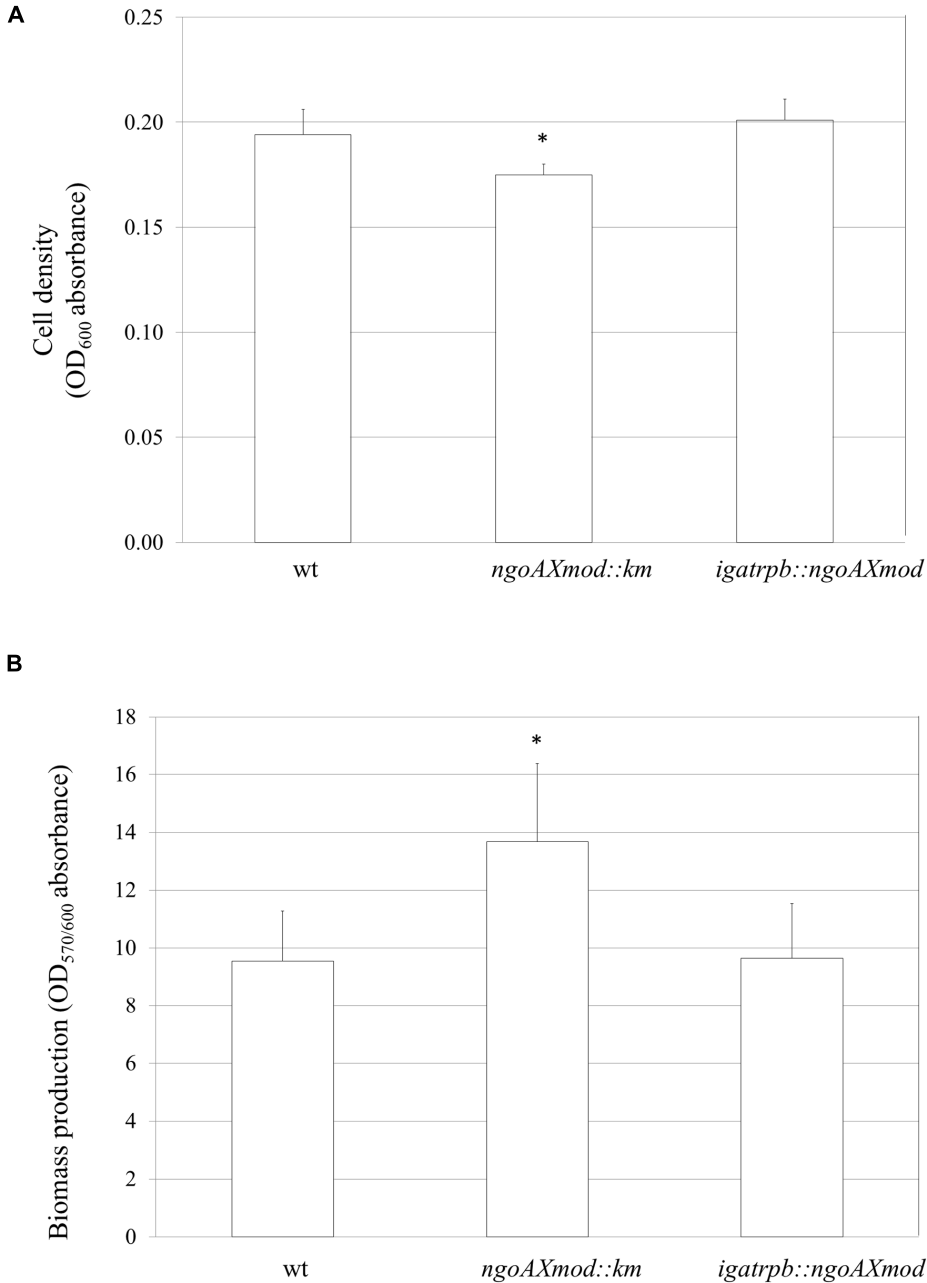
**Growth and biofilm biomass of *ngoAXmod::km* and *igatrpb::ngoAXmod N. gonorrhoeae* mutants compared to the wt strain.** Measurements were performed by crystal violet staining after a 24-h growth without shaking. **(A)** Biofilm and planktonic cell growth was measured at absorbance OD_600_; **(B)** Biomass production: ratio of cells that forms biofilm (OD_570_) versus total grown cells (OD_600_). Asterisks represent statistical significant differences in comparison to the wt strain (*P* < 0.05).

Gonococci under study were examined for their growth and adherence abilities to polystyrene surfaces. After a 24-h growth without shaking, the overall density OD_600_, reflecting both planktonic and biofilm-engaged gonococcal cells, was determined. The amount of *N. gonorrhoeae ngoAXmod::km* cells was slightly yet significantly lower (OD_600_ = 0.175 ± 0.005) than gonococcal wt cells (OD_600_ = 0.194 ± 0.012; *P* < 0.05). Reintroduction of the wt copy of *ngoAXmod* resulted in a similar amount of complementary mutant cells (OD_600_ = 0.201 ± 0.010) as compared to the wt strain (*P* > 0.05) (**Figure 3A**). Subsequently, after intensive washing of cells not engaged in biofilm formation, we measured the proportion of biofilm-forming cells by staining them with crystal violet. The NgoAX knock-out strain formed a slightly larger biofilm biomass per cell than the wt strain (OD_570/600_ = 13.8 ± 2.24 and 9.35 ± 2.06 respectively; *P* < 0.05) as determined by comparing the biofilm biomass to the total amount of cells (**Figure 3B**). Introduction of the wt copy of *ngoAXmod* reduced biofilm formation at the level of wt strain: OD_570/600_ = 9.64 ± 1.89.

The ability of *N. gonorrhoeae* FA1090 wt, *ngoAXmod::km* and complementation mutant to form biofilm was also evaluated using FE SEM and SCLM.

**FIGURE 4:**
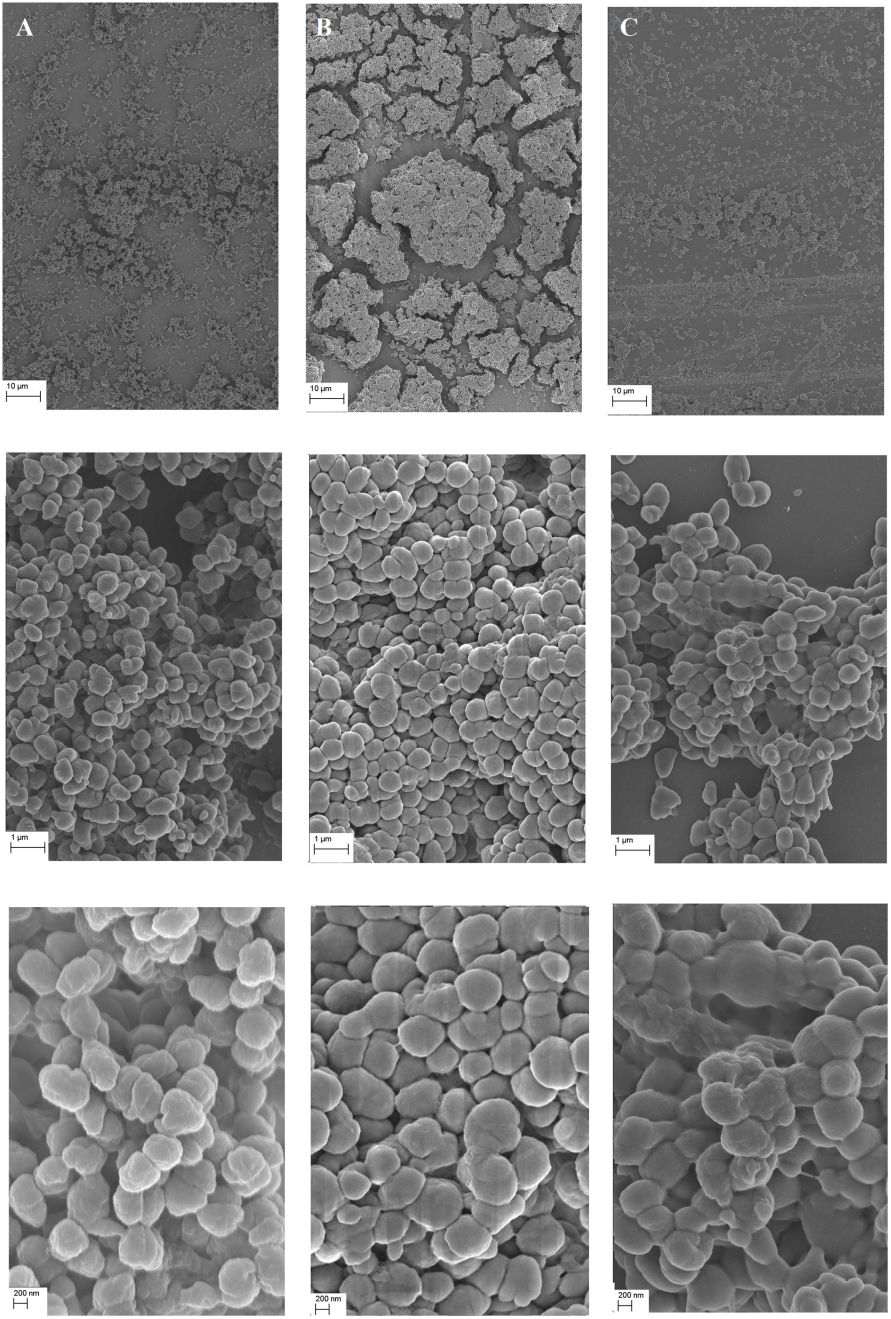
**Biofilm produced by *N. gonorrhoeae* on cover glass after 24 h visualized by Field Emission Scanning Electron Microscopy. (A)** Biofilm formed by the wt FA1090 strain; **(B)** Biofilm formed by the *N. gonorrhoeae ngoAXmod::km* mutant and **(C)** Biofilm formed by the *N. gonorrhoeae igatrpb::ngoAXmod* mutant. Experiments were triplicated and representative photographs are shown.

For FE SEM, bacteria were grown under standard growth conditions on a cover slip glass for 24 h. As shown on **Figure 4A**, biofilm formed by the wt *N. gonorrhoeae* was evenly spread on the whole area of the glass plates with cells associated in layers. The biofilm was observed to have a structural roughness. Gonococcal cells seemed to be attached to each other in a dispersed, relaxed way and exhibited characteristic for gonococci, membrane vesicles (blebbings) on their surface and had coffee-bean shape. These observations were in consistency with those from our previous work (Kwiatek et al., 2014).

In contrast, biofilm formed by the *N. gonorrhoeae* NgoAX knock-out mutant was more compact with closely associated cells forming compact clumps on the glass surface (**Figure 4B**). The cell shape seemed similar to that of the wt, yet slightly more rounded. However, the surface of the cells was different: mutant cells with interrupted *ngoAXmod* gene were smooth, without blebbings characteristic for the wt *Neisseria*. Moreover, the mutant cells seemed to be positioned more closely to each other than wt cells. The formed biofilm was more opulent. Complementation of the *ngoAXmod*-encoded function restored the wt feature of the biofilm: it was more dispersed than the one formed by the *ngoAXmod::km* mutant. Moreover, cells presented blebbings on their surface, characteristic for the wt strain (**Figure 4C**).

**FIGURE 5:**
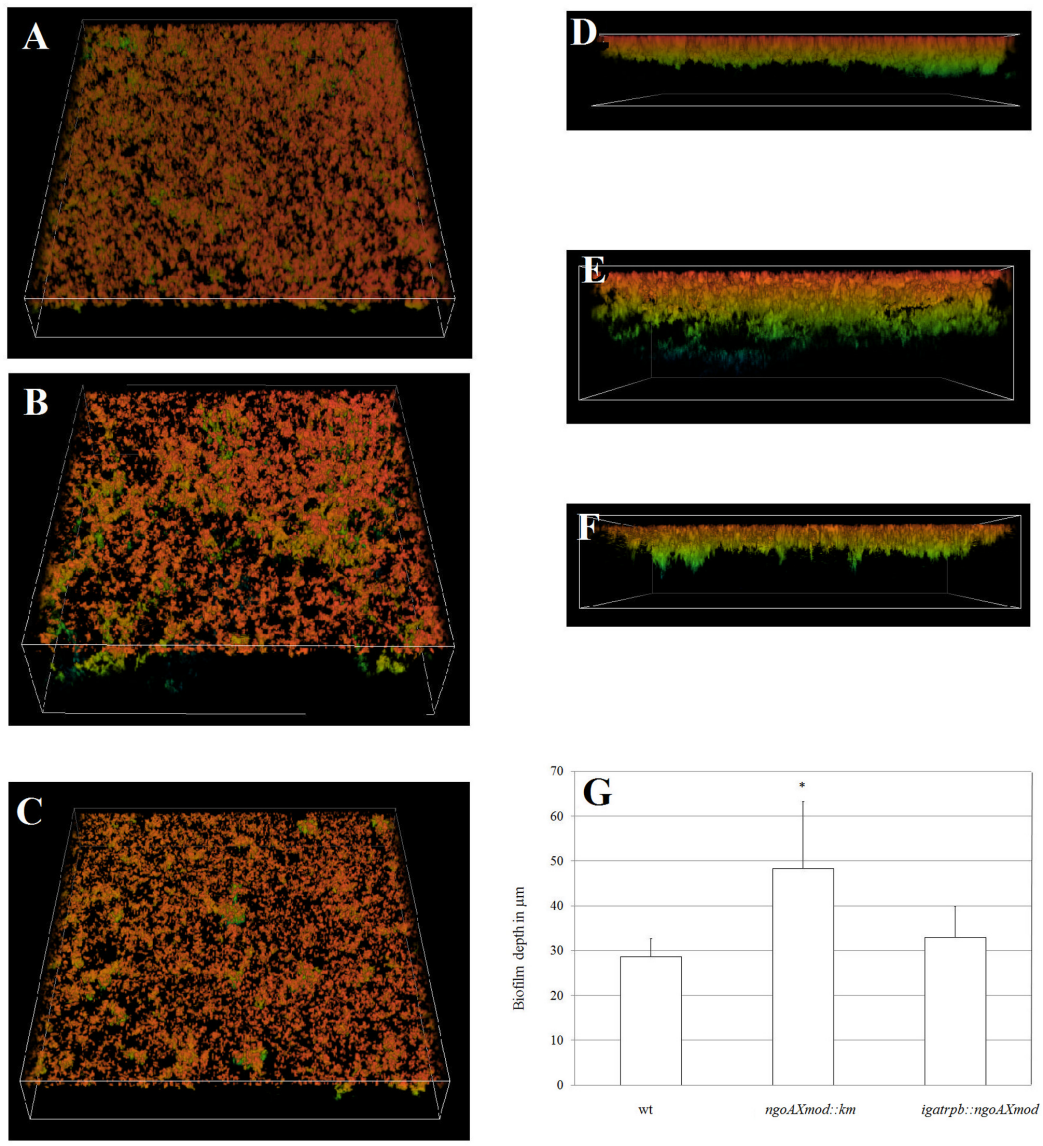
**Three-dimensional biofilm structures visualized by SCLM produced by *N. gonorrhoeae* after 24 h. (A–C)** Three-dimensional biofilm structures and density; **(D–F)** biofilm thickness. **(A)** and **(D)** biofilm produced by the wt strain; **(B)** and **(E)** biofilm produced by the gonococcal *ngoAXmod::km* mutant; **(C)** and **(F)** biofilm produced by the gonococcal *igatrpb*::*ngoAXmod* mutant **(G)** biofilm thickness in μm as measured by the NIS-ELEMENTS software. Experiments were triplicated and representative images are shown.

To study live gonococcal biofilm SCLM was applied. For this purpose, *N. gonorrhoeae* was cultivated on glass plates and after a 24-h growth cells were stained by acridine orange. As shown on **Figures 5A,D** biofilm formed by the wt strain was dense and uniformly distributed on the glass surface. On the other side, the biofilm formed by the gonococcal mutant in the gene encoding M.NgoAX was more relaxed, dispersed but thicker (**Figures 5B,E**). The wt feature of the biofilm was recovered in the complementation mutant (**Figures 5C,F**). We also measured the thickness of each biofilm. This examination revealed that the thickest biofilm was in case of the *ngoAXmod::km* strain: 48.3 (±14.9) μm (**Figure 5G**), while the wt strain produced a biofilm with 28.6 (±4.0) μm. Finally, the biofilm produced by the *N. gonorrhoeae* complementation mutant in the *ngoAXmod* gene resembled the one formed by the wt strain: 32.9 (±6.9) μm. The difference in thickness of the biofilm formed by the NgoAX knock-out mutant and wt strain was statistically significant (*P <*0.05).

### Interaction of *N. gonorrhoeae* with Human Epithelial Cells Hec-1-B is Modulated by M.NgoAX Expression

**FIGURE 6:**
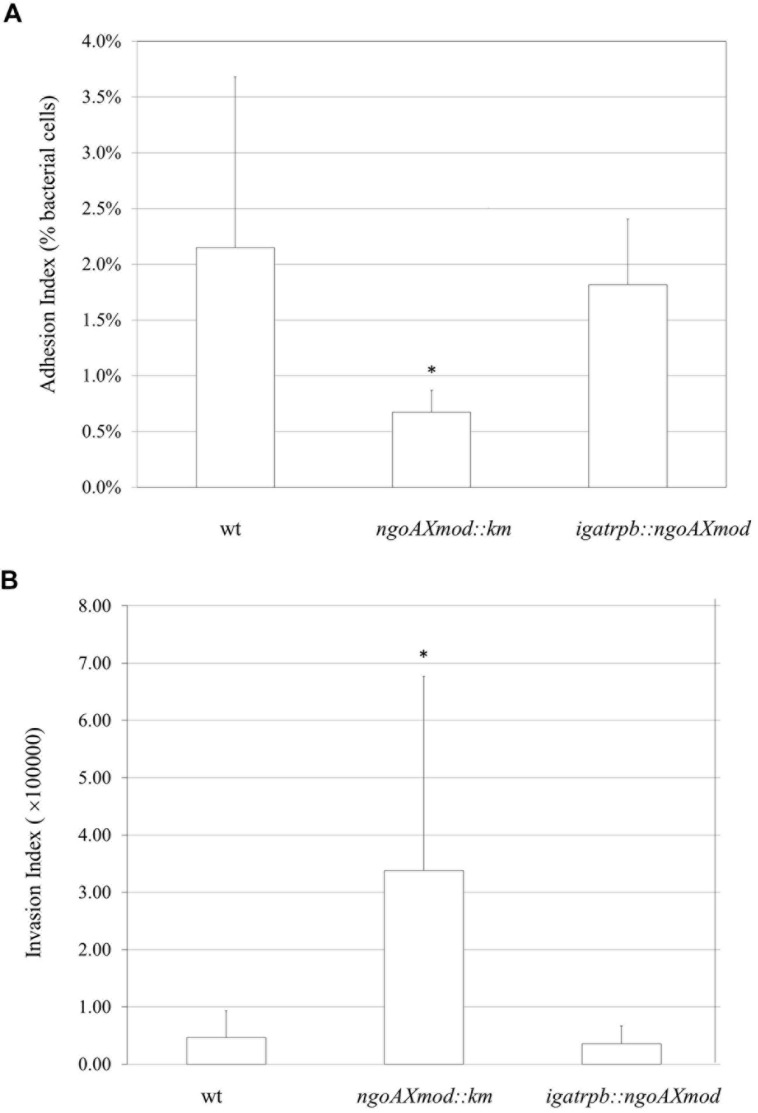
***Neisseria gonorrhoeae* adhesion and invasion of human Hec-1-B cells.** Human epithelial cells were infected for 4 h with the gonococcal *ngoAXmod::km* mutant, wt strain or *igatrpb:: ngoAXmod* mutant. **(A)** The adhesion index was calculated as a ratio between the numbers of bacteria that adhered to human cells to the total number of cells used for infection. **(B)** The invasion index was calculated by dividing the number of gentamicin resistant CFU by the adhesion index. The asterisk represents the statistical difference in comparison to the wt strain (*P* < 0.05).

After demonstrating that the gonococcal NgoAX knock-out mutant strain differs from the wt strain in adherence to glass or polystyrene surfaces, we investigated its ability to attach and invade epithelial cells. Monolayers of human Hec-1-B cells were infected with gonococci for 4 h. Then, bacteria that were attached to or have invaded into the epithelial cells were released. We also enumerated total bacterial cells added to the Hec-1-B cells (total CFU). Under our experimental conditions, the adhesion index of the wt strain was 2.15 (±1.53) (**Figure 6A**). The gonococcal *ngoAXmod::km* mutant had almost a three-times lower adhesion compared to the wt strain (**Figure 6A**). The adhesion index determined for the *ngoAXmod::km* strain was 0.672 (±0.2; *P* < 0.05). Adhesion of the complementation mutant seemed similar to that of the wt type with the adhesion index 1.82 (±0.59). Yet, the difference between the *igatrpb::ngoAXmod* strain and wt strain was not statistically significant (*P* > 0.05).

Using the gentamycin resistance assay we also established the invasion index, which allows to determine the amount of gonococcal cells that penetrate into the host human cells. As seen on **Figure 6B**, in our experimental conditions, the invasion index for wt gonococcal cells was 4.67 (±3.09) × 10^4^. Inactivation of the M.NgoAX methyltransferase enhanced almost 10-fold the penetration of mutant gonococci cells into human host cells: 3.38 (±0.93) × 10^5^ (*P* < 0.05). Complementation of the *ngoAXmod* gene restored the level of invasion to 3.58 (±1.89) × 10^4^, which was not statistically different from that noted for the wt cells (*P* > 0.05).

## Discussion

We studied the impact of M.NgoAX methyltransferase inactivation on the overall gene expression and fitness of *N. gonorrhoeae* FA1090. For this purpose, we constructed two mutant strains: one, with inactivated *ngoAXmod* gene by insertion of the *km* cassette (the NgoAX knock-out mutant) and a second, in which this mutation was complemented by insertion of the wt *ngoAXmod* allele into a non-coding intergenic region between *iga* and *trpb* chromosomal genes (Ramsey et al., 2012). Inactivation of the M.NgoAX methyltransferase mimics phase variation of the *ngoAXmod* gene in *N. gonorrhoeae* cells. Indeed, the *ngoAXmod* gene was designated as a candidate for phase variation (Snyder et al., 2001; Srikhanta et al., 2009) and we have previously observed that the restriction phenotype associated with the NgoAX RM system can switch randomly (Adamczyk-Poplawska et al., 2009). This transition may be involved in a mechanism that increases gonococcal evasion strategies or environmental adaptation.

Inactivation of type III M.NgoAX methyltransferase studied in this work had a huge impact on cell fitness and resulted in deregulation of expression of 121 genes at least twofold compared to the wt strain under standard growth conditions. In contrast, it was demonstrated that inactivation of the M.NgoAXII type III methyltransferase encoded by *ngoAXIImod* did not influence gene expression under standard gonococcal growth conditions and the cut-off threshold set at 1.5 fold (Srikhanta et al., 2009). A small pool of 54 genes was deregulated by M.NgoAXII inactivation when *N. gonorrhoeae* FA1090 was cultivated under iron-limiting conditions (Srikhanta et al., 2009). Different effects of methyltransferases on gene expression may be explained by variations in their expression levels. As demonstrated by qRT-PCR, the *ngoAXmod* gene, encoding M.NgoAX, is sevenfold more highly expressed in the wt *N. gonorrhoeae* FA1090 than *ngoAXIImod*, encoding M.NgoAXII. Srikhanta et al. (2009) have compared the amino acid sequences of type III methyltransferases present in *N. gonorrhoeae* and *meningitidis* strains. Phylogenetic studies revealed that *Neisseriaceae* have two distinct *mod* genes: *modA* and *modB*. All studied *N. gonorrhoeae* strains possess the same *modB1* gene but different *modA* alleles. *N. gonorrhoeae* FA1090 has *modB1* gene (*ngoAXmod*), encoding M.NgoAX and *modA13* (*ngoAXIImod*), encoding M.NgoAXII. The conservation of *modB1* gene among strains suggests that proteins encoded by this gene have a general role. Due to, we postulated, that M.NgoAX is the main epigenetic regulator in *N. gonorrhoeae* FA1090. Phase variation of gene encoding M.NgoAXII plays a role in regulation of phasevarion in particular growth conditions such as iron-depleted conditions. Our hypothesis is reinforced by the fact that methylation by M.NgoAXII is not detected in *N. gonorrhoeae* FA1090 genome (PacBio results, Rebase).

Lack of methylation by NgoAX affected not only gene expression. Also changes in bacterial cell growth were observed. Mutant cells with inactivated M.NgoAX methyltransferase were able to grow more rapidly when cultivated in liquid medium with shaking and without interaction with a solid support. During growth without shaking, the number of NgoAX knock-out gonococci increased at a lower rate than wt *Neisseria*. This suggested a role of M.NgoAX methyltransferase in growth control of *N. gonorrhoeae* FA1090 depending of growth conditions. Differences in growth abilities of the studied strains were also reflected in biofilm formation.

Biofilms provide a number of advantages when it comes to bacterial survival and pathogenesis. Biofilm formation by gonococci may promote their ability to persist as an asymptomatic infection in the female genital tract (Greiner et al., 2005; Bozicevic et al., 2006). Adhesion studies of the NgoAX knock-out strain to polystyrene plates or human epithelial cells indicated that the mutant strain adhered more poorly to both supports than the wt FA1090. Surprisingly, however, a greater amount of mutant gonococci were engaged in biofilm formation as determined by microplate assay and biofilm biomass/cell measurements or by FE SEM or SCLM observations. All assays confirmed an increase of mutant gonococcal cells involved in biofilm structure compared to the wt strain. However, there seemed to be fewer cell to cell interactions in case of the NgoAX knock-out mutant than for the wt gonococci. The observation of live bacterial biofilm by confocal microscopy indicated that the biofilm formed by NgoAX knock-out cells was thicker and more relaxed compared to the wt strain. Similar changes in biofilm structure were observed for mutants in which the other type III methyltransferase, M.NgoAXII, was deleted. Three-dimensional images of the biofilms showed that the *ngoAXIImod::km* (*modA13::kan*) FA1090 strain formed a thick and dense biofilm, while the wt biofilm was thinner with few sparse patches of cells scattered across the attachment surface (Srikhanta et al., 2009).

Biofilm FE SEM observations, which demanded different sample preparations, showed distortion of the biofilm, most probably due to a large cell-free space at the base of the biofilm structure formed by the NgoAX knock-out mutant cells, and collapsing of the biofilm structure. Altogether, the gathered data suggest that NgoAX methylation on one side increases the planktonic cell division rate and by this augments the number of cells that may be engaged in biofilm formation, and on the other, decreases the cell-cell or cell-support adhesion. In *in vivo* conditions phase variation of NgoAX methyltransferase expression may tightly regulate this process.

Phillips et al. (2012) used stable isotope labeling by amino acids in cell culture (SILAC method) to compare gene expression in biofilm-forming and planktonic *N. gonorrhoeae* 1291 strain. In this study, nitrite reductase was detected as the key enzyme required for anaerobic growth – conditions favoring biofilm formation. The *ngo1276* (*aniA*) gene, encoding this enzyme, was shown to be upregulated in the *N. gonorrhoeae* FA1090 NgoAX knock-out mutant as demonstrated by expression microarrays assay. The biofilm of the mutant was shown to consist of a greater number of bacteria than wt. In comparison to proteomes of planktonic organisms, Phillips et al. (2012) demonstrated that 73 proteins were upregulated and 54 downregulated. Nearly a third of the upregulated proteins were involved in energy metabolism, while cell envelope proteins represented the second largest group. In our study, we observed in the mutant strain deregulation of 13 genes encoding proteins directly involved in energy production and conversion, such as the previously mentioned *aniA* gene or genes coding for NADH-quinone oxidoreductase (*ngo1737* and *ngo1748*, respectively).

To study the mechanism of gonococcal biofilm formation, Falsetta et al. (2009) compared gene expression in biofilms and planktonic *N. gonorrhoeae* 1291 cells. The study showed that 3.8% of the genome of the biofilm-forming strain was differentially regulated at least twofold compared to planktonic cells. Genes that were highly upregulated in biofilms included, among others, *aniA*, and *ccp* genes. These genes encode enzymes involved in the anaerobic respiratory metabolism and stress tolerance. It was also noted that *aniA* and *ccp* insertional mutants were attenuated for biofilm development on glass, and on transformed primary human cervical cells. We found that expression of these genes (*ngo1276* and *ngo1769*, respectively) was also upregulated in the NgoAX knock-out mutant. These data suggest that biofilm formation during infection of epithelial cells by *N. gonorrhoeae* may be associated with the control of nitric oxide steady-state levels (Falsetta et al., 2009).

The changes in biofilm structure and the ability to penetrate into human epithelial cells can reflect the facility of *N. gonorrhoeae* to efficient adaptation to different environments during the gonococcal infection. Indeed, the overall adhesion of mutant bacteria to the human host was lower than adhesion of wt gonococci; yet, *ngoAXmod::km* cells were more numerous inside human epithelial Hec-1-B cells than wt bacteria. These results suggest that NgoAX knock-out cells have less ability to attach to human cells, but have a greater facility to penetrate inside the host cells. Adherence to and invasion of host epithelial cells are the first steps of infection initiated by *N. gonorrhoeae* in humans. Therefore, it is suggested that phase variation of the *ngoAXmod* gene may have a physiological role in dissemination of *N. gonorrhoeae*. Also functional studies using *N. gonorrhoeae* FA1090 confirmed that *ngoAXIImod* ON and OFF strains have distinct phenotypes in biofilm formation, in antimicrobial resistance or in infection of primary human cells (Srikhanta et al., 2009).

Interaction of gonococci to the epithelial cells has been extensively studied and shown to be based on the interaction of several bacterial virulence factors, for example pili, porin or Opa, to their respective receptors (Edwards and Apicella, 2004). In the *N. gonorrhoeae* FA1090 *ngoAXmod* knock-out, we found many genes encoding proteins with predicted outer membrane functions^2^ or which belong to the COG M (cell wall/membrane/envelop biogenesis) category. Among them, expression of genes, encoding proteins with predicted outer membrane localization, *ngo1513* (encoding OpaD), *ngo2093* (encoding FetA), *ngo2121*(encoding lipoprotein VacJ), *ngo0021*, *ngo0510*, *ngo0555* and *ngo0868* (encoding OpcA), was deregulated both in the studied NgoAX knock-out mutant (Supplementary Table S2) and biofilm-forming gonococci versus planktonic cells (Phillips et al., 2012). Moreover, several other proteins, which gene expression was affected in the *ngoAXmod::km* mutant cells, are suspected to be involved in cell adhesion. This includes *ngo1068*, *ngo1393* and *ngo0180* genes, which encode Maf adhesins. The Maf family comprises adhesins with multiple silent loci, which bind to glycolipids on host cell genes (Paruchuri et al., 1990). *LgtE* (*ngo2159*), encoding lacto-*N*-neotetraose biosynthesis glycosyl transferase, and *rfaC* (*ngo1934)*, encoding ADP-heptose–LPS heptosyltransferase, genes encode enzymes that are involved in lipooligosacharride (LOS) synthesis. Their expression is downregulated in the *ngoAXmod::km* gonococci. Oligosaccharide extension of LOS can be involved in receptor-mediated interactions. It was demonstrated that LOS containing lacto-*N*-neotetraose facilitates invasion but not the adherence of *N. gonorrhoeae* to the human cervical epidermal carcinoma ME180 cell line (Song et al., 2000). Moreover, the terminal lactosamine of lacto-*N*-neotetraose play an important role in invasion of primary urethral epithelial cells (Harvey et al., 2001). The downexpression of the *lgtE* gene in NgoAXP knock-out mutant cells may result in control of the lacto-*N*-neotetraose biosynthesis, which may be necessary for Hec-1-B cell invasion by gonococci. A naturally occurring variation of the terminal carbohydrates on the LOS molecule correlates with altered disease states and may reflect the adaptation of *Neisseria* to different steps of infection (John et al., 1999). Moreover, the LOS transport across the periplasmic space and their exposition at the cell surface seemed to be enhanced in NgoAX knock-out cells by a more intensive expression of *lgtF* (*ngo1228*) and *lgtG* (*ngo1229)* genes, encoding LgtF and LgtG transporters, respectively.

Our results also showed downregulation of genes involved in pili formation. The expression of *ngo0095* (*pilP*), *ngo0096* (*pilO*), *ngo0097* (*pilN*) and *ngo0098* (*pilM*) was deregulated in the NgoAX knock-out mutant. Type IV pili are another element responsible for the first steps of bacterial adhesion, but account also for bacterial aggregation (i.e., microcolony formation). Weaker expression of the respective genes may contribute to a more relaxed feature of the biofilms. For *N. meningitidis*, immunofluorescent staining with antipilus antibodies showed that meningococcal piliation dramatically reduces at later time points of bacterial cell interaction with human cells (Pujol et al., 1997).

The most downregulated gene determined in our study, excluding *ngo0545* (*ngoAXmod*) was *ngo0574*, coding for carbonic anhydrase (Cah), which catalyzes the conversion of carbon dioxide to bicarbonate and protons. Deregulation of this gene was demonstrated also by proteomic analysis of the biofilm (Phillips et al., 2012), and transcriptomic analysis of type III RM mutant (Srikhanta et al., 2009), but not by transcriptomic analysis of the biofilm (Falsetta et al., 2009). The role of this enzyme in biofilm formation should be investigated as *N. gonorrhoeae* FA1090 possesses two Cah genes and the second one (*ngo2079*) was not deregulated in NgoAX knock-out cells. Bacterial Cah proteins are speculated to be involved in the critical steps of bacterial life cycle, including stages important for survival, invasion, and pathogenicity (Supuran, 2011). It seems that Cah activity may be necessary for biofilm formation and may also be involved in cell to host adhesion.

We also observed deregulation of genes encoded by prophages present in the *N. gonorrhoeae* chromosome. *N. gonorrhoeae* FA1090 contains several prophages in its genome (Piekarowicz et al., 2006, 2007). Five of them are dsDNA phages (Φ1–5) and 4 are ssDNA phages (Φ6–9). In the NgoAX knock-out mutant, 14 genes of Φ1 phage, 4 genes from Φ2 phage and two from Φ5 phage were deregulated. Moreover, 2 genes from Φ8 ssDNA phage were shown to be downregulated. The role of proteins encoded by these deregulated genes remains unknown. Yet, it is known that viral proteins maybe involved in bacterial fitness and pathogenesis. For example, proteins encoded by prophages found in *Streptococcus mitis* (Bensing et al., 2001) or *Enterococcus faecalis* (Matos et al., 2013) were necessary for adhesion of these bacteria to human platelets, which is the first step toward the development of infective endocarditis. Further studies should be performed to investigate the role of prophage-encoded proteins in *N. gonorrhoeae* pathogenesis.

Regulation of gene expression by methylation of specific DNA sequences by Dam or type III methyltransferases has been reviewed (Wion and Casadesús, 2006; Marinus and Casadesus, 2009; Srikhanta et al., 2010). As in case of Dam methyltransferase, the target site methylation state may affect DNA binding by the regulatory protein, which directly regulates transcription or even acts on RNA polymerase affinity to DNA. The widespread distribution of phase variable RM systems in host-adapted pathogenic bacteria, such as *H. influenzae* or *N. gonorrhoeae*, suggests the existence of a mechanism coordinating the switch of multiple gene expression. The interactions between RNA polymerase with DNA are randomly affected by a different methylation pattern. Such strategy may be used to generate cells specialized distinctly in human cell invasion or biofilm formation (Srikhanta et al., 2009).

Our data suggest that the M.NgoAX methyltransferase may be involved in *N. gonorrhoeae* pathogenicity, specifically in control of biofilm formation, adhesion to host cells and invasion of epithelial cells. Phase variation of *ngoAXmod* gene expression may be necessary for changing the gonococcal abilities in the first step of human cell invasion.

## Conflict of Interest Statement

The authors declare that the research was conducted in the absence of any commercial or financial relationships that could be construed as a potential conflict of interest.

## Footnotes

1 http://www.ncbi.nlm.nih.gov/geo/query/acc.cgi?acc=GSE71703

2 http://db.psort.org/

## Abbreviations

COG: clusters of orthologous groups
FE SEM: Field Emission Scanning Electron Microscopy
km: kanamycin cassette
RM: restriction modification
SCLM: Scanning Confocal Laser Microscopy
wt: wild-type

## References

[B1] Adamczyk-Poplawska M. Lower M. Piekarowicz A. (2009). Characterization of the NgoAXP: phase-variable type III restriction-modification system in *Neisseria gonorrhoeae*. *FEMS Microbiol. Lett.* 300 25–35. 10.1111/j.1574-6968.2009.01760.x 19758331

[B2] Arber W. (2000). Genetic variation: molecular mechanisms and impact on microbial evolution. *FEMS Microbiol. Rev.* 24 1–7. 10.1111/j.1574-6976.2000.tb00529.x 10640595

[B3] Bayliss C. D. Callaghan M. J. Moxon E. R. (2006). High allelic diversity in the methyltransferase gene of a phase variable type III restriction-modification system has implications for the fitness of *Haemophilus influenzae*. *Nucleic Acids Res.* 34 4046–4059. 10.1093/nar/gkl568 16914439 PMC1557822

[B4] Bensing B. A. Siboo I. R. Sullam P. M. (2001). Proteins PblA and PblB of *Streptococcus mitis*, which promote binding to human platelets, are encoded within a lysogenic bacteriophage. *Infect. Immun.* 69 6186–6192. 10.1128/IAI.69.10.6186-6192.2001 PMC98750 11553559

[B5] Bozicevic I. Fenton K. A. Martin I. M. Rudd E. A. Ison C. A. Nanchahal K. (2006). Epidemiological correlates of asymptomatic gonorrhea. *Sex. Transm. Dis.* 33 289–295. 10.1097/01.olq.0000194582.44222.c9 16554697

[B6] Broadbent S. E. Davies M. R. van der Woude M. W. (2010). Phase variation controls expression of *Salmonella* lipopolysaccharide modification genes by a DNA methylation-dependent mechanism. *Mol. Microbiol.* 77 337–353. 10.1111/j.1365-2958.2010.07203.x 20487280 PMC2909390

[B7] De Bolle X. Bayliss C. D. Field D. van de Ven T. Saunders N. J. Hood D. W. (2000). The length of a tetranucleotide repeat tract in *Haemophilus influenzae* determines the phase variation rate of a gene with homology to type III DNA methyltransferases. *Mol. Microbiol.* 35 211–222. 10.1046/j.1365-2958.2000.01701.x 10632891

[B8] Dillard J. P. (2006). Genetic manipulation of *Neisseria gonorrhoeae*. *Curr. Protoc. Microbiol.* 4A2.1–4A2.19. 10.1002/9780471729259.mc04a02s00 18770590

[B9] Dryden D. T. Murray N. E. Rao D. N. (2001). Nucleoside triphosphate-dependent restriction enzymes. *Nucleic Acids Res.* 29 3728–3741. 10.1093/nar/29.18.3728 PMC55918 11557806

[B10] Dziewit L. Adamczuk M. Szuplewska M. Bartosik D. (2011). DIY series of genetic cassettes useful in construction of versatile vectors specific for Alphaproteobacteria. *J. Microbiol. Methods* 86 166–174. 10.1016/j.mimet.2011.04.016 21569803

[B11] Edgar R. Domrachev M. Lash A. E. (2002). Gene Expression Omnibus: NCBI gene expression and hybridization array data repository. *Nucleic Acids Res.* 30 207–210. 10.1093/nar/30.1.207 11752295 PMC99122

[B12] Edwards J. L. Apicella M. A. (2004). The molecular mechanisms used by *Neisseria gonorrhoeae* to initiate infection differ between men and women. *Clin. Microbiol. Rev.* 17 965–981. 10.1128/CMR.17.4.965-981.2004 15489357 PMC523569

[B13] Edwards J. L. Butler E. K. (2011). The pathobiology of *Neisseria gonorrhoeae* lower female genital tract infection. *Front. Microbiol.* 2:102 10.3389/fmicb.2011.00102 PMC3129011 21747805

[B14] Falsetta M. L. Bair T. B. Ku S. C. Vanden Hoven R. N. Steichen C. T. McEwan A. G. (2009). Transcriptional profiling identifies the metabolic phenotype of gonococcal biofilms. *Infect. Immun.* 77 3522–3532. 10.1128/IAI.00036-09 19528210 PMC2737991

[B15] Fox K. L. Dowideit S. J. Erwin A. L. Srikhanta Y. N. Smith A. L. Jennings M. P. (2007a). *Haemophilus influenzae* phasevarions have evolved from type III DNA restriction systems into epigenetic regulators of gene expression. *Nucleic Acids Res.* 35 5242–5252. 10.1093/nar/gkm571 17675301 PMC1976455

[B16] Fox K. L. Srikhanta Y. N. Jennings M. P. (2007b). Phase variable type III restriction-modification systems of host-adapted bacterial pathogens. *Mol. Microbiol.* 65 1375–1379. 10.1111/j.1365-2958.2007.05873.x 17714447

[B17] Greiner L. L. Edwards J. L. Shao J. Rabinak C. Entz D. Apicella M. A. (2005). Biofilm formation by *Neisseria gonorrhoeae*. *Infect. Immun.* 73 1964–1970. 10.1128/IAI.73.4.1964-1970.2005 PMC1087446 15784536

[B18] Harvey H. A. Jennings M. P. Campbell C. A. Williams R. Apicella M. A. (2001). Receptor-mediated endocytosis of *Neisseria gonorrhoeae* into primary human urethral epithelial cells: the role of the asialoglycoprotein receptor. *Mol. Microbiol.* 42 659–672. 10.1046/j.1365-2958.2001.02666.x 11722733

[B19] Holmes K. K. (1999). *Sexually Transmitted Diseases.* New York, NY: McGraw-Hill Health Professions Division Publisher.

[B20] Hopper S. Wilbur J. S. Vasquez B. L. Larson J. Clary S. Mehr I. J. (2000). Isolation of *Neisseria gonorrhoeae* mutants that show enhanced trafficking across polarized T84 epithelial monolayers. *Infect. Immun.* 68 896–905. 10.1128/IAI.68.2.896-905.2000 10639460 PMC97219

[B21] Hümbelin M. Suri B. Rao D. N. Hornby D. P. Eberle H. Pripfl T. (1988). Type III DNA restriction and modification systems EcoP1 and EcoP15. *Nucleotide sequence* of the EcoP1 operon, the EcoP15 mod gene and some EcoP1 mod mutants. *J. Mol. Biol.* 200 23–29. 10.1016/0022-2836(88)90330-0 2837577

[B22] Ison C. A. (2011). “Biology of *Neisseria gonorrhoeae* and the clinical picture of infection,” in *Sexually Transmitted Infections and Sexually Transmitted Diseases*, eds Gross G. E. Tyring S. K. (Heidelberg: Springer), 77–90. 10.1007/978-3-642-14663-3_6

[B23] Jarvis G. A. Chang T. L. (2012). Modulation of HIV transmission by *Neisseria gonorrhoeae*: molecular and immunological aspects. *Curr. HIV Res.* 10 211–217. 10.2174/157016212800618138 22384840 PMC4149178

[B24] Jeltsch A. (2003). Maintenance of species identity and controlling speciation of bacteria: a new function for restriction/modification systems? *Gene* 317 13–16. 10.1016/S0378-1119(03)00652-8 14604787

[B25] John C. M. Schneider H. Griffiss J. M. (1999). *Neisseria gonorrhoeae* that infect men have lipooligosaccharides with terminal N-acetyllactosamine repeats. *J. Biol. Chem.* 274 1017–1025. 10.1074/jbc.274.2.1017 9873046

[B26] Jordan P. W. Snyder L. A. Saunders N. J. (2005). Strain-specific differences in *Neisseria gonorrhoeae* associated with the phase variable gene repertoire. *BMC Microbiol.* 5:21 10.1186/1471-2180-5-21 PMC1097732 15857514

[B27] Kobayashi I. (2001). Behavior of restriction-modification systems as selfish mobile elements and their impact on genome evolution. *Nucleic Acids Res.* 29 3742–3756. 10.1093/nar/29.18.3742 11557807 PMC55917

[B28] Kwiatek A. Bacal P. Wasiluk A. Trybunko A. Adamczyk-Poplawska M. (2014). The dam replacing gene product enhances *Neisseria gonorrhoeae* FA1090 viability and biofilm formation. *Front. Microbiol.* 5:712 10.3389/fmicb.2014.00712 PMC4269198 25566225

[B29] Low D. A. Weyand N. J. Mahan M. J. (2001). Roles of DNA adenine methylation in regulating bacterial gene expression and virulence. *Infect. Immun.* 69 7197–7204. 10.1128/IAI.69.12.7197-7204.2001 11705888 PMC98802

[B30] Malott R. J. Keller B. O. Gaudet R. G. McCaw S. E. Lai C. C. Dobson-Belaire W. N. (2013). *Neisseria gonorrhoeae*-derived heptose elicits an innate immune response and drives HIV-1 expression. *Proc. Natl. Acad. Sci. U.S.A.* 110 10234–10239. 10.1073/pnas.1303738110 23733950 PMC3690901

[B31] Marinus M. G. Casadesus J. (2009). Roles of DNA adenine methylation in host-pathogen interactions: mismatch repair, transcriptional regulation, and more. *FEMS Microbiol. Rev.* 33 488–503. 10.1111/j.1574-6976.2008.00159.x 19175412 PMC2941194

[B32] Marrazzo J. M. Handsfield H. H. Sparling P. F. (2010). “Neisseria gonorrhoeae,” in *Mandell, Douglas, and Bennett’s Principles and Practice of Infectious Diseases*, eds Mandell G. L. Bennett J. E. Dolin R. (Philadelphia, PA: Churchill Livingstone/Elsevier), 2753–2770. 10.1016/B978-0-443-06839-3.00212-5

[B33] Matos R. C. Lapaque N. Rigottier-Gois L. Debarbieux L. Meylheuc T. Gonzalez-Zorn B. (2013). *Enterococcus faecalis* prophage dynamics and contributions to pathogenic traits. *PLoS Genet.* 9:e1003539 10.1371/journal.pgen.1003539 PMC3675006 23754962

[B34] Mruk I. Kobayashi I. (2014). To be or not to be: regulation of restriction-modification systems and other toxin-antitoxin systems. *Nucleic Acids Res.* 42 70–86. 10.1093/nar/gkt711 23945938 PMC3874152

[B35] Natale D. A. Galperin M. Y. Tatusov R. L. Koonin E. V. (2000). Using the COG database to improve gene recognition in complete genomes. *Genetica* 108 9–17. 10.1023/A:1004031323748 11145426

[B36] Paruchuri D. K. Seifert H. S. Ajioka R. S. Karlsson K. A. So M. (1990). Identification and characterization of a *Neisseria gonorrhoeae* gene encoding a glycolipid-binding adhesin. *Proc. Natl. Acad. Sci. U.S.A.* 87 333–337. 10.1073/pnas.87.1.333 2153292 PMC53257

[B37] Phillips N. J. Steichen C. T. Schilling B. Post D. M. Niles R. K. Bair T. B. (2012). Proteomic analysis of *Neisseria gonorrhoeae* biofilms shows shift to anaerobic respiration and changes in nutrient transport and outermembrane proteins. *PLoS ONE* 7:e38303 10.1371/journal.pone.0038303 PMC3368942 22701624

[B38] Piekarowicz A. Kłyz A. Majchrzak M. Adamczyk-Popławska M. Maugel T. K. Stein D. C. (2007). Characterization of the dsDNA prophage sequences in the genome of *Neisseria gonorrhoeae* and visualization of productive bacteriophage. *BMC Microbiol.* 7:66 10.1186/1471-2180-7-66 PMC1931599 17615066

[B39] Piekarowicz A. Majchrzak M. Kłyz A. Adamczyk-Popławska M. (2006). Analysis of the filamentous bacteriophage genomes integrated into *Neisseria gonorrhoeae* FA1090 chromosome. *Pol. J. Microbiol.* 55 251–260. 17416061

[B40] Price C. Bickle T. A. (1986). A possible role for DNA restriction in bacterial evolution. *Microbiol. Sci.* 3 296–299. 2856420

[B41] Pujol C. Eugène E. de Saint Martin L. Nassif X. (1997). Interaction of *Neisseria meningitidis* with a polarized monolayer of epithelial cells. *Infect. Immun.* 65 4836–4842. 9353073 10.1128/iai.65.11.4836-4842.1997 PMC175694

[B42] Ramsey M. E. Hackett K. T. Kotha C. Dillard J. P. (2012). New complementation constructs for inducible and constitutive gene expression in *Neisseria gonorrhoeae* and *Neisseria meningitidis*. *Appl. Environ. Microbiol.* 78 3068–3078. 10.1128/AEM.07871-11 22327577 PMC3346468

[B43] Rao D. N. Dryden D. T. Bheemanaik S. (2014). Type III restriction-modification enzymes: a historical perspective. *Nucleic Acids Res.* 42 45–55. 10.1093/nar/gkt616 23863841 PMC3874151

[B44] Roberts R. J. Vincze T. Posfai J. Macelis D. (2015). REBASE–a database for DNA restriction and modification: enzymes, genes and genomes. *Nucleic Acids Res.* 43 D298–D299. 10.1093/nar/gku1046 25378308 PMC4383893

[B45] Ryan K. A. Lo R. Y. (1999). Characterization of a CACAG pentanucleotide repeat in *Pasteurella haemolytica* and its possible role in modulation of a novel type III restriction-modification system. *Nucleic Acids Res.* 27 1505–1511. 10.1093/nar/27.6.1505 10037813 PMC148345

[B46] Sambrook J. Russell D. W. (2001). *Molecular Cloning: A Laboratory Manual*, 3rd Edn, Vol. 1 Cold Spring Harbor, NY: Cold Spring Harbor Laboratory Press

[B47] Seib K. L. Jen F. E. Tan A. Scott A. L. Kumar R. Power P. M. (2015). Specificity of the ModA11, ModA12 and ModD1 epigenetic regulator N(6)-adenine DNA methyltransferases of *Neisseria meningitidis*. *Nucleic Acids Res.* 43 4150–4162. 10.1093/nar/gkv219 25845594 PMC4417156

[B48] Sneppen K. Semsey S. Seshasayee A. S. Krishna S. (2015). Restriction modification systems as engines of diversity. *Front. Microbiol.* 6:528 10.3389/fmicb.2015.00528 PMC4451750 26082758

[B49] Snyder L. A. Butcher S. A. Saunders N. J. (2001). Comparative whole-genome analyses reveal over 100 putative phase-variable genes in the pathogenic *Neisseria* spp. *Microbiology* 147 2321–2332. 10.1099/00221287-147-8-2321 11496009

[B50] Song W. Ma L. Chen R. Stein D. C. (2000). Role of lipooligosaccharide in Opa-independent invasion of *Neisseria gonorrhoeae* into human epithelial cells. *J. Exp. Med.* 191 949–960. 10.1084/jem.191.6.949 10727457 PMC2193109

[B51] Srikhanta Y. N. Dowideit S. J. Edwards J. L. Falsetta M. L. Wu H. J. Harrison O. B. (2009). Phasevarions mediate random switching of gene expression in pathogenic *Neisseria*. *PLoS Pathog.* 5:e1000400 10.1371/journal.ppat.1000400 PMC2667262 19390608

[B52] Srikhanta Y. N. Fox K. L. Jennings M. P. (2010). The phasevarion: phase variation of type III DNA methyltransferases controls coordinated switching in multiple genes. *Nat. Rev. Microbiol.* 8 196–206. 10.1038/nrmicro2283 20140025

[B53] Srikhanta Y. N. Gorrell R. J. Steen J. A. Gawthorne J. A. Kwok T. Grimmond S. M. (2011). Phasevarion mediated epigenetic gene regulation in *Helicobacter pylori*. *PLoS ONE* 6:e27569 10.1371/journal.pone.0027569 PMC3230613 22162751

[B54] Srikhanta Y. N. Maguire T. L. Stacey K. J. Grimmond S. M. Jennings M. P. (2005). The phasevarion: a genetic system controlling coordinated, random switching of expression of multiple genes. *Proc. Natl. Acad. Sci. U.S.A.* 102 5547–5551. 10.1073/pnas.0501169102 15802471 PMC556257

[B55] Stein D. C. Gunn J. S. Radlinska M. Piekarowicz A. (1995). Restriction and modification systems of *Neisseria gonorrhoeae*. *Gene* 157 19–22. 10.1016/0378-1119(94)00649-D 7607490

[B56] Stepanovic S. Vukovic D. Dakic I. Savic B. Svabic-Vlahovic M. (2000). A modified microtiter-plate test for quantification of staphylococcal biofilm formation. *J. Microbiol. Methods* 40 175–179. 10.1016/S0167-7012(00)00122-6 10699673

[B57] Supuran C. T. (2011). Carbonic anhydrase inhibitors and activators for novel therapeutic applications. *Future Med. Chem.* 3 1165–1180. 10.4155/fmc.11.69 21806379

[B58] Swanson J. (1978). Studies on gonococcus infection. XII. Colony color and opacity variants of gonococci. *Infect. Immun.* 19 320–331. 415006 10.1128/iai.19.1.320-331.1978 PMC414083

[B59] Tatusov R. L. Galperin M. Y. Natale D. A. Koonin E. V. (2000). The COG database: a tool for genome-scale analysis of protein functions and evolution. *Nucleic Acids Res.* 28 33–36. 10.1093/nar/28.1.33 10592175 PMC102395

[B60] Unemo M. Nicholas R. A. (2012). Emergence of multidrug-resistant, extensively drug-resistant and untreatable gonorrhea. *Future Microbiol.* 7 1401–1422. 10.2217/fmb.12.117 23231489 PMC3629839

[B61] Vasu K. Nagaraja V. (2013). Diverse functions of restriction-modification systems in addition to cellular defense. *Microbiol. Mol. Biol. Rev.* 77 53–72. 10.1128/MMBR.00044-12 23471617 PMC3591985

[B62] WHO (2012). *Global Action Plan to Control the Spread and Impact of Antimicrobial Resistance in Neisseria gonorrhoeae.* Geneva: World Health Organization Press.

[B63] Williams R. J. (2003). Restriction endonucleases: classification, properties, and applications. *Mol. Biotechnol.* 23 225–243. 10.1385/MB:23:3:225 12665693

[B64] Wion D. Casadesús J. (2006). N6-methyl-adenine: an epigenetic signal for DNA-protein interactions. *Nat. Rev. Microbiol.* 4 183–192. 10.1038/nrmicro1350 16489347 PMC2755769

