# Supplementary material for: Type III Methyltransferase M.NgoAX from Neisseria gonorrhoeae FA1090 Regulates Biofilm Formation and Interactions with Human Cells
Source: Front Microbiol. 2015 Dec 21;6:1426. doi: 10.3389/fmicb.2015.01426 (PMC4685087; doi:10.3389/fmicb.2015.01426)
Supplement: Supplementary file 1 [file Table_1.DOCX]

**Table S1. List of primers used for gene amplification or qRT-PCR.** For qRT-PCR primers, primer names are the same as gene accession number.

| **Primers for amplification of *ngo0545* from *N. gonorrhoeae* FA1090 wt and mutant strains** | | | |
| --- | --- | --- | --- |
| **Name** | **Forward primer** | **Reverse primer** | **Product size bp** |
| Smamod/Nhemod | TCCCCCGGGCACCCTGATAATCACATTCAAC | CTAGCTAGCACCGAGGATTTTAAAATGCTGCCAACC | see in text |
| **Primers for amplification of** pMPMigatrpBopaCM | | | |
| **Name** | **Forward primer** | **Reverse primer** | **Product size bp** |
| Smatrpb/Nheiga | TCCCCCGGGGACTAACAAAGCTACAGCTCAATGC | CTAGCTAGCGTCCGAAGCATGCATGGAATGAC | 6578 |
| **Primers for amplification of *iga-trpb* interregion** | | | |
| Iga/Trpb | TATGTGGCCGGCGATATTGG | AAAGCGCAGATGCAGGAAGC | see in text |
| **Primers for amplification of fragments of *ngo0545* or *ngo0545::km* genes** | | | |
| **Name** | **Forward primer** | **Reverse primer** | **Product size bp** |
| AXPleft/ AXPright | TTGAAGCCGGAATCGCCCGTGTAATC | TACAAGCCGAAATTGCGCCACAGACC | see in text |
| **Primers for qRT-PCR** | | | |
| **Primers for internal standard** | | | |
| **Name** | **Forward primer** | **Reverse primer** | **Product size bp** |
| 16SRNA | gcgtgggtagcaaacaggat | CGCGTTAGCTACGCTACCAAG | 81 |
| **Primers for the study of *ngo0545::km* mutant gene expression** | | | |
| **Name** | **Forward primer** | **Reverse primer** | **Product size bp** |
| Ngo0097 | CGAGCTGTCGGAAATACAAA | ATTCAGGCTGTCGAGGATTT | 121 |
| Ngo0135 | CGAAATCTGGCTCAAACAAA | CTCCATGGTCAAATCGTCAG | 79 |
| Ngo0395 | GTTTCGTCGATACCGTGATG | ATATAAACCGTGGCAAACGC | 91 |
| Ngo0574 | GTCCTTCCATCTGGAACGTCA | CGGCAAAGCGGTAGTATTTC | 71 |
| Ngo0806 | TTCATCTTCCCGTCGGTTAT | CCTGAAATTTCCGCCATATC | 146 |
| Ngo0868 | ACTTTCTGGGTAAAGGCGAA | TGAATATCGTCAAACGGCAT | 136 |
| Ngo0869 | ATCCTGCATATCGACCAACA | AAGGGCGTAACAATCAGACC | 113 |
| Ngo0908 | AGTACCGAAGCAGAAATCCG | ATTTCCTGCCCTATTGATGC | 98 |
| Ngo1046 | CGAATTTGAAGAACGCTTGA | GCCGACCAAAGTATGGATTT | 97 |
| Ngo1156 | GGGTAATCAGCTTGTCCGAT | GGTCGAATTTAGCGACAACA | 122 |
| Ngo1192 | TCTTGGCTGCAACAGCTTAC | TCGGCAATCAAATCCAAATA | 101 |
| Ngo1993 | TCATCGTTTCGCAGCTTG | GAGGACGACTTCGCGTTC | 92 |
| Ngo1205 | AACTGGTATTTCACGCCACA | GTGCGTACAGTTCTTGCGTT | 85 |
| Ngo1276 | GCACTTCCACATTCAGCTTC | GTACATACCGTTGGCGATGT | 98 |
| Ngo1455 | GGCGATGCTCAAAGTAACAA | GGCATCCTGCCTGATTATTT | 88 |
| Ngo1648 | CAAAGACACCGCATCAGTTC | CGCTTGTCCCTGATTGTATG | 116 |
| Ngo1728 | TACAAAGTCGCCGAATTCAC | GCGGTAAAGGCGTTCATATC | 110 |
| Ngo2090 | CGTCAAAGGAGATTGGGACT | GGTCAGCGTTTGGAAGAGTT | 112 |
| Ngo2092 | GCCTGCCGTATTTAGAGGAA | TCAATTTGTCAAACGCCTTG | 141 |
| Ngo2093 | ACCAACATCGTTACGCTTCA | GTCGACAGAGTTCTGACCCA | 150 |
| Ngo2121 | CTGTTTATCCGCCCAAGAAT | GCGCGTGTAGCTGTATTTGT | 149 |
| Ngo1513 | CGGCAACCAGCTTAACATAA | GGGTTTGAATTTGTCGTTGA | 124 |
| Ngo1771 | GAAAGAAACGGTGGTTCCC | CGTGGTCGACATCAATCTGT | 91 |
| **Primers for the study of *ngo0641* gene expression** | | | |
| Ngo0641 | CCAATACATCGGCATTGAAC | CATAAACAAACTCGCCACCA | 134 |
