# Supplementary material for: Type III Methyltransferase M.NgoAX from Neisseria gonorrhoeae FA1090 Regulates Biofilm Formation and Interactions with Human Cells
Source: Front Microbiol. 2015 Dec 21;6:1426. doi: 10.3389/fmicb.2015.01426 (PMC4685087; doi:10.3389/fmicb.2015.01426)
Supplement: Supplementary file 2 [file Table_2.DOCX]

**Table S2. Differentially expressed genes in the NgoAXP knock-out *N. gonorrhoeae* mutant (*ngo0545::km*) versus wild-type *N. gonorrhoeae* FA1090.** Identity of the genes is indicated by gene and protein accession numbers, according to NCBI in the annotation of the *N. gonorrhoeae* FA1090 genome. For microarray and qRT-PCR values, the presented average ratio is the mean of *N. gonorrhoeae drg::cm* mutant: wild type *N. gonorrhoeae* FA1090. Only those genes with an expression ratio above 1.5-fold and *P* < 0.05 were included in this study. Protein functions were assigned according to BLAST and Uniprot databases. COGs were assigned with the NCBI Conserved domains server (http://www.ncbi.nlm.nih.gov/Structure/cdd/wrpsb.cgi). NF – known conserved domain not found. NC – not confirmed.

| **Gene name** | **Uniprot** | **function** | **Microarray** | **qRTPCR** | **Cog symbol** | **category** |
| --- | --- | --- | --- | --- | --- | --- |
| **down** |  |  |  |  |  |  |
| *ngo0021* | Q5FAL3 | TonB-dependent receptor protein, iron related | -1.78 |  | COG4773 | P |
| *ngo0022* | Q5FAL2 | hypothetical protein | -1.52 |  | COG0662 | G |
| *ngo0025* | Q5FAK9 | AraC family transcriptional regulator | -2.21 |  | COG2207 | K |
| *ngo0076* | Q5FAF0 | hypothetical protein | -2.08 |  | COG2819 | R |
| *ngo0095* | Q5FAD1 | type IV pilus assembly protein PilP | -1.96 |  | COG3168 | N, U |
| *ngo0096* | Q5FAD0 | type IV pilus assembly protein PilO | -1.84 |  | COG3167 | S |
| *ngo0097* | Q5FAC9 | pilus assembly protein PilN | -2.08 | -1.49 | COG3166 | N, U |
| *ngo0098* | Q5FAC8 | type IV pilus assembly ATPase PilM | -1.63 |  | COG4972 | N, U |
| *ngo0115* | Q5FAB2 | hypothetical protein | -1.99 |  | NF |  |
| *ngo0158* | Q5FA72 | pepN, aminopeptidase | -1.60 |  | COG0308 | E |
| *ngo0167* | Q5FA64 | hypothetical protein | -2.12 |  | NF |  |
| *ngo0215* | Q5FA19 | iron ABC transporter ATP-binding protein FbpC | -1.56 |  | COG3842 | E |
| *ngo0261* | Q5F9X5 | N-(5'-phosphoribosyl)anthranilate isomerase | -1.80 |  | COG0135 | E |
| *ngo0292* | Q5F9U7 | hypothetical protein | -1.77 |  | COG2010 | C |
| *ngo0322* | Q5F9R8 | hypothetical protein | -1.51 |  | COG3467 | V |
| *ngo0389* | Q5F9K4 | pseudouridylate synthase | -1.96 |  | COG1187 | J |
| *ngo0432* | Q5F9G3 | hypothetical protein | -2.53 |  | NF |  |
| *ngo0465* | Q5F9D4 | phage associated protein | -1.71 |  | NF |  |
| *ngo0468* | Q5F7P3 | phage associated protein | -1.51 |  | NF |  |
| *ngo0486* | Q5F7R1 | hypothetical protein, phage associated? | -1.84 |  | NF |  |
| *ngo0494* | Q5F9A5 | phage associated protein | 2.85 |  | NF |  |
| *ngo0498* | Q5F9A1 | phage associated protein | -2.46 |  | NF |  |
| *ngo0500* | Q5F999 | phage associated protein | -1.56 |  | NF |  |
| *ngo0503* | Q5F996 | phage associated protein | -2.79 |  | NF |  |
| *ngo0531* | Q5F968 | hypothetical protein | -2.18 |  | NF |  |
| *ngo0545* | Q5F958 | type III restriction-modification system methyltransferase | -64.93 |  | COG2189 | L |
| *ngo0555* | Q5F948 | hypothetical protein | -3.35 |  | NF |  |
| *ngo0571* | Q5F933 | hypothetical protein | -1.68 |  | COG4942 | O |
| *ngo0574* | Q5F930 | carbonic anhydrase | -10.21 | -12.50 | COG3338 | P |
| *ngo0633* | Q5F8X6 | iron-sulfur cluster assembly scaffold protein | -1.97 |  | COG0822 | C |
| *ngo0639* | Q5F8X1 | L-lactate dehydrogenase | -1.91 |  | COG1304 | C |
| *ngo0640* | Q5F8X0 | type III restriction modification system endonuclease | -2.20 |  | COG3421 | V |
| *ngo0641* | Q5F8W9 | type III restriction modification system methylase | -2.42 |  | COG2189 | L |
| *ngo0699* | Q5F8R4 | type I restriction-modification system specificity protein | -2.52 |  | COG0732 | V |
| *ngo0720* | Q5F8P7 | hypothetical protein | -1.62 |  | NF |  |
| *ngo0721* | Q5F8P6 | phage associated protein | -1.62 |  | NF |  |
| *ngo0725* | Q5F8P2 | phage associated protein | -4.19 |  | NF |  |
| *ngo0765* | Q5F8K7 | arsenate reductase family protein | -1.51 |  | COG1393 | P |
| *ngo0776* | Q5F8J7 | hypothetical protein | -1.52 |  | NF |  |
| *ngo0786* | Q5F8I7 | uracil-DNA glycosylase | -1.55 |  | COG0692 | L |
| *ngo0806* | Q5F8G7 | hypothetical protein | -2.14 |  | COG1648 | H |
| *ngo0827* | Q5F8F0 | hypothetical protein | -1.51 |  | NF |  |
| *ngo0868* | Q5F8B8 | OpcA-Class 5 outer membrane protein | -2.13 |  | NF |  |
| *ngo0888* | Q5F8A0 | hypothetical protein | -1.53 |  | NF |  |
| *ngo0908* | Q5F881 | antitoxin FitA | -2.16 | -1.50 | COG4691 | S |
| *ngo0915* | Q5F876 | dihydrolipoamide dehydrogenase | -1.96 |  | COG1249 | C |
| *ngo0919* | Q5F872 | hypothetical protein | -1.67 |  | COG2938 | O |
| *ngo0926* | Q5F865 | peroxiredoxin family protein/glutaredoxin | -1.51 |  | COG0678 | O |
| *ngo0967* | Q5F834 | hypothetical protein | -1.91 |  | NF |  |
| *ngo0982* | Q5F819 | hypothetical protein | -2.47 |  | NF |  |
| *ngo0991* | Q5F812 | succinyl-diaminopimelate desuccinylase | -1.54 |  | COG0624 | E |
| *ngo0998* | Q5F806 | dnaG, synthesizes RNA primers at the replication forks, DNA primase | -1.76 |  | COG0358 | L |
| *ngo0999* | Q5F805 | RNA polymerase sigma factor RpoD | -1.88 |  | COG0568 | K |
| *ngo1014* | Q5F7Z0 | hypothetical protein | -2.30 |  | NF |  |
| *ngo1031* | Q5F7Y0 | single-stranded DNA binding protein | -1.57 |  | COG0629 | L |
| *ngo1034* | Q5F7X7 | hypothetical protein | -1.74 |  | NF |  |
| *ngo1046* | Q5F7W9 | ATP-dependent chaperone ClpB | -2.15 | -2.12 | COG0542 | O |
| *ngo1049* | Q5F7W6 | hypothetical protein | -1.68 |  | COG5266 | P |
| *ngo1051* | Q5F7W5 | hypothetical protein | -2.14 |  | NF |  |
| *ngo1062* | Q5F7V5 | hypothetical protein | -1.50 |  | NF |  |
| *ngo1068* | Q5F7U9 | MafB protein | -1.57 |  | NF |  |
| *ngo1069* | Q5F7U8 | hypothetical protein | -2.16 |  | NF |  |
| *ngo1104* | Q5F7R6 | phage associated protein | -2.43 |  | NF |  |
| *ngo1147* | Q5F7M3 | hypothetical protein | -2.58 |  | NF |  |
| *ngo1156* | Q5F7L5 | hypothetical protein | -2.01 | -1.53 | COG3468 | M, U |
| *ngo1158* | Q5F7L3 | hypothetical protein | -1.83 |  | NF |  |
| *ngo1169* | Q5F7A9 | phage associated protein | -1.58 |  | NF |  |
| *ngo1170* | Q5F7A8 | phage associated protein | -2.18 |  | NF |  |
| *ngo1189* | Q5F7I8 | hslO, Heat shock protein 33 (Hsp33): Cytosolic protein that acts as a molecular chaperone under oxidative conditions | -1.60 |  | COG1281 | O |
| *ngo1199* | Q5F7H8 | ATP dependent DNA helicase | -1.55 |  | COG1643 | L |
| *ngo1210* | Q5F7G8 | hypothetical protein | -1.87 |  | COG1943 | L |
| *ngo1345* | Q5F742 | 3-octaprenyl-4-hydroxybenzoate carboxy-lyase | -1.57 |  | COG0043 | H |
| *ngo1393* | Q5F6H4 | adhesin MafA | -1.91 |  | NF |  |
| *ngo1396* | Q5F6Z3 | oxidoreductase | -1.66 |  | COG0644 | C |
| *ngo1399* | Q5F6Z0 | ABC transporter ATP-binding protein | -1.60 |  | COG1131 | V |
| *ngo1444* | Q5F6V1 | hypothetical protein | -1.52 |  | NF |  |
| *ngo1479* | Q5F6R9 | hypothetical protein | -1.77 |  | COG0784 | T |
| *ngo1482* | Q5F6R7 | hypothetical protein | -1.53 |  | COG2830 | S |
| *ngo1613* | Q5F6E6 | integrase/recombinase | -1.85 |  | COG0582 | L |
| *ngo1635* | Q5F6C4 | hypothetical protein | -1.60 |  | NF |  |
| *ngo1637* | Q5F6C2 | hypothetical protein | -2.53 |  | NF |  |
| *ngo1648* | Q5F6B2 | transposase, Putative invertase related gene 7, putative phage associated protein | -2.10 | -1.52 | COG3547 | L |
| *ngo1708* | Q5F658 | dinG, helicase involved in DNA repair and perhaps also replication | -1.68 |  | COG1199 | L |
| *ngo1748* | Q5F618 | NADH-quinone oxidoreductase subunit D | -1.57 |  | COG0649 | C |
| *ngo1755* | Q5F611 | hypothetical protein | -1.67 |  | NF |  |
| *ngo1771* | Q5F5Z7 | membrane protein | -4.02 |  | COG0668 | M |
| *ngo1846* | Q5F5S0 | hypothetical protein | -1.61 |  | NF |  |
| *ngo1847* | Q5F5R9 | hypothetical protein | -2.26 |  | NF |  |
| *ngo1856* | Q5F5R0 | nusG, Modulates Rho-dependent transcription termination | -1.66 |  | COG0250 | K |
| *ngo1897* | Q5F5M4 | dTDP-glucose 4,6-dehydratase | -1.57 |  | COG1088 | M |
| *ngo1913* | Q5F5L1 | hypothetical protein | -1.64 |  | NF |  |
| *ngo1924* | Q5F5K0 | arsenate reductase | -1.55 |  | COG1393 | C |
| *ngo1933* | Q5F5J1 | amidase, nicotinamidase | -1.55 |  | COG1335 | Q |
| *ngo1934* | Q5F5J0 | ADP-heptose--LPS heptosyltransferase | -1.64 |  | COG0859 | M |
| *ngo1955* | Q5F5H1 | hypothetical protein | -1.78 |  | COG2911 | S |
| *ngo1959* | Q5F5G7 | hypothetical protein | -1.54 |  | NF |  |
| *ngo1977* | Q5F5F1 | 5-formyltetrahydrofolate cyclo-ligase | -2.48 |  | COG0212 | H |
| *ngo2006* | Q5F5C3 | thiamine biosynthesis protein ThiS | -1.64 |  | COG2104 | H |
| *ngo2007* | Q5F5C2 | thiamin-phosphate pyrophosphorylase | -1.88 |  | COG0352 | H |
| *ngo2051* | Q5F579 | deferrochelatase/peroxidase | -1.65 |  | COG2837 | P |
| *ngo2074* | Q5F562 | ubiquinone biosynthesis O-methyltransferase | -1.68 |  | COG2227 | Q |
| *ngo2079* | Q5F557 | carbonic anhydrase | -1.53 |  | COG0288 | P |
| *ngo2092* | Q5F544 | iron ABC transporter substrate-binding protein | -2.08 | NC | COG4607 | P |
| *ngo2093* | Q5F543 | Fet, TonB-dependent siderophore receptor | -3.63 | -2.63 | COG1629 | P |
| *ngo2159* | Q5F4Y4 | lacto-N-neotetraose biosynthesis glycosyl transferase, LOS biosynthesis | -1.52 |  | COG3306 | M |
| *ngo2176* | Q5F4W8 | hypothetical protein | -1.60 |  | NF |  |
| **up** |  |  |  |  |  |  |
| *ngo0015* | Q5FAJ9 | hypothetical protein | 4.20 |  | NF |  |
| *ngo0020* | Q5FAJ4 | hypothetical protein | 2.09 |  | COG0607 | P |
| *ngo0050* | Q5FAH0 | endonuclease | 1.53 |  | COG2852 | L |
| *ngo0053* | Q5FAG9 | carbamoyl phosphate synthase small subunit | 1.55 |  | COG0505 | F |
| *ngo0110* | Q5FAB6 | magnesium/citrate transporter | 3.21 |  | COG2851 | C |
| *ngo0113* | Q5FAB4 | ribonuclease G | 1.62 |  | COG1530 | J |
| *ngo0127* | Q5FAA1 | rbn-UPF0761 membrane protein | 1.94 |  | COG1295 | S |
| *ngo0135* | Q5FA94 | beta-hexosaminidase | 2.78 | 2.64 | COG1472 | G |
| *ngo0136* | Q5FA93 | Na+/H+ antiporter | 2.01 |  | COG1757 | C |
| *ngo0137* | Q5FA92 | hypothetical protein | 2.85 |  | NF |  |
| *ngo0138* | Q5FA91 | serine protease | 1.55 |  | COG0265 | O |
| *ngo0141* | Q5FA88 | hypothetical protein | 2.61 |  | NF |  |
| *ngo0145* | Q5FA84 | endoribonuclease | 2.35 |  | COG0319 | R |
| *ngo0147* | Q5FA82 | hypothetical protein | 3.02 |  | COG0644 | C |
| *ngo0160* | Q5FA71 | hypothetical protein | 2.21 |  | NF |  |
| *ngo0161* | Q5FA70 | hypothetical protein | 2.19 |  | NF |  |
| *ngo0166* | Q5FA65 | hypothetical protein | 2.11 |  | NF |  |
| *ngo0180* | Q5FA52 | Maf-like protein | 1.71 |  | COG0424 | D |
| *ngo0181* | Q5FA51 | sec-independent protein translocase protein TatC | 1.76 |  | COG0805 | U |
| *ngo0195* | Q5FA39 | polyamine ABC transporter permease | 1.65 |  | COG1176 | E |
| *ngo0196* | Q5FA38 | polyamine ABC transporter permease | 1.53 |  | COG1177 | E |
| *ngo0264* | Q5F9X2 | bacteriocin production protein | 1.70 |  | COG1286 | S |
| *ngo0285* | Q5F9V3 | hypothetical protein | 1.55 |  | COG1714 | S |
| *ngo0323* | Q5F9R7 | 2-amino-4-hydroxy-6-hydroxymethyldihydropteridine pyrophosphokinase | 1.76 |  | COG0801 | H |
| *ngo0324* | Q5F9R6 | hypothetical protein | 1.74 |  | COG3094 | S |
| *ngo0364* | Q5F9M9 | restriction endonuclease | 2.31 |  | NF |  |
| *ngo0386* | Q5F9K7 | cystathionine gamma-synthase | 1.59 |  | COG0626 | E |
| *ngo0395* | Q5F9J8 | multidrug efflux protein | 2.07 | 3.00 | COG0534 | V |
| *ngo0407* | Q5F9I7 | type I restriction-modification system endonuclease | 2.27 |  | COG0610 | V |
| *ngo0427* | Q5F9G7 | peptidase | 2.67 |  | COG3271 | R |
| *ngo0430* | Q5F9G4 | membrane protein | 2.13 |  | NF |  |
| *ngo0448* | Q5F9E9 | hypothetical protein | 2.03 |  | NF |  |
| *ngo0478* | Q5F9C1 | phage associated protein | 1.56 |  | NF |  |
| *ngo0490* | Q5F9A9 | phage associated protein | 2.48 |  | NF |  |
| *ngo0495* | Q5F9A4 | phage associated protein, Phage terminase large subunit | 2.57 |  | COG5362 | X |
| *ngo0510* | Q5F989 | phage associated protein | 3.99 |  | NF |  |
| *ngo0513* | Q5F986 | phage associated protein, Phage terminase large subunit | 2.13 |  | COG5362 | X |
| *ngo0522* | Q5F977 | phage associated protein | 1.65 |  | COG5281 | S |
| *ngo0523* | Q5F976 | phage associated protein | 1.59 |  | NF |  |
| *ngo0529* | Q5F970 | choline/carnitine/betaine transport protein | 4.47 |  | COG1292 | M |
| *ngo0534* | Q5F965 | alanine export protein | 2.17 |  | COG1696 | M |
| *ngo0602* | Q5F907 | MerR family transcriptional regulator | 2.78 |  | COG0789 | K |
| *ngo0606* | Q5F903 | sodium-dependent transport protein | 2.84 |  | COG0733 | R |
| *ngo0649* | Q5F8W1 | hypothetical protein | 4.05 |  | NF |  |
| *ngo0672* | Q5F8T8 | hypothetical protein | 2.10 |  | NF |  |
| *ngo0674* | Q5F8T6 | 3-isopropylmalate dehydrogenase | 1.56 |  | COG0473 | E |
| *ngo0696* | Q5F8R7 | recombination factor protein RarA | 1.66 |  | COG2256 | L |
| *ngo0712* | Q5F8Q5 | hypothetical protein | 2.16 |  | NF |  |
| *ngo0723* | Q5F8P4 | hypothetical protein | 2.08 |  | COG1598 | S |
| *ngo0724* | Q5F8P3 | phage associated protein | 2.42 |  | NF |  |
| *ngo0735* | Q5F8N4 | hypothetical protein | 2.48 |  | NF |  |
| *ngo0736* | Q5F8N3 | hypothetical protein | 2.63 |  | NF |  |
| *ngo0797* | Q5F8H6 | DNA-binding protein | 2.68 |  | COG1396 | K |
| *ngo0843* | Q5F8D9 | hypothetical protein | 1.88 |  | NF |  |
| *ngo0850* | Q5F8D3 | proA-Catalyzes the phosphorylation of L-glutamate during the proline biosynthesis pathway, Gamma-glutamyl phosphate reductase | 1.55 |  | COG0014 | E |
| *ngo0852* | Q5F8D1 | membrane protein | 2.04 |  | COG3235 | S |
| *ngo0854* | Q5F8C9 | hypothetical protein | 2.63 |  | NF |  |
| *ngo0869* | Q5F8B7 | hypothetical protein | 2.54 | 1.54 | COG0586 | S |
| *ngo0897* | Q5F891 | hypothetical protein | 2.17 |  | NF |  |
| *ngo0903* | Q5F886 | aminoglycoside resistance efflux transporter protein | 2.36 |  | COG0477 | G, E, P, R |
| *ngo0942* | Q5F850 | membrane protein | 1.54 |  | COG2839 | S |
| *ngo0953* | Q5F844 | hypothetical protein | 5.48 |  | NF |  |
| *ngo0960* | Q5F838 | hypothetical protein | 1.61 |  | COG1179 | H |
| *ngo0979* | Q5F822 | hypothetical protein | 1.88 |  | NF |  |
| *ngo1003* | Q5F801 | hypothetical protein | 1.50 |  | COG3649 | L |
| *ngo1027* | Q5F7Y4 | hypothetical protein | 2.26 |  | NF |  |
| *ngo1041* | Q5F7X4 | exopolyphosphatase | 1.56 |  | COG0248 | P, F |
| *ngo1086* | Q5F7T4 | phage associated protein | 1.58 |  | NF |  |
| *ngo1093* | Q5F7S7 | phage associated protein | 2.23 |  | NF |  |
| *ngo1101* | Q5F7R9 | phage associated protein | 2.93 |  | NF |  |
| *ngo1132* | Q5F7N8 | phage associated protein, Helix-Turn-Helix DNA binding domain of transcription regulators from the MerR superfamily | 2.13 |  | COG3311 | K, X |
| *ngo1159* | Q5F7L2 | hypothetical protein | 3.12 |  | NF |  |
| *ngo1191* | Q5F7I6 | hypothetical protein | 3.95 |  | NF |  |
| *ngo1192* | Q5F7I5 | murein hydrolase transporter LrgA | 3.06 | 4.42 | COG1380 | R |
| *ngo1193* | Q5F7I4 | murein hydrolase transporter LrgB | 2.73 |  | COG1346 | M |
| *ngo1198* | Q5F7H9 | hypothetical protein | 1.73 |  | COG2194 | R |
| *ngo1203* | Q5F7H5 | trpD-Anthranilate phosphoribosyltransferase | 1.52 |  | COG0547 | E |
| *ngo1205* | Q5F7H3 | TonB-dependent receptor protein | 3.06 | 2.72 | COG1629 | P |
| *ngo1206* | Q5F7H2 | phosphatidylserine decarboxylase | 1.82 |  | COG0688 | I |
| *ngo1212* | Q5F7G6 | pyrG-CTP synthase, catalyzes the ATP-dependent amination of UTP to CTP with either L-glutamine or ammonia as the source of nitrogen | 1.66 |  | COG0504 | F |
| *ngo1213* | Q5F7G5 | long-chain-fatty-acid--CoA ligase | 1.70 |  | COG0318 | I |
| *ngo1221* | Q5F7F7 | GntR family transcriptional regulator | 1.75 |  | COG1802 | K |
| *ngo1228* | Q5F7F0 | LPS ABC transporter permease LptF | 1.75 |  | COG0795 | R |
| *ngo1229* | Q5F7E9 | LPS ABC transporter permease LptG | 1.61 |  | COG0795 | R |
| *ngo1237* | Q5F7E1 | hypothetical protein | 1.53 |  | COG1999 | O |
| *ngo1245* | Q5F7D3 | membrane protein | 1.68 |  | COG1132 | V |
| *ngo1248* | Q5F7D0 | hypothetical protein | 4.83 |  | NF |  |
| *ngo1251* | Q5F7C7 | hypothetical protein | 2.50 |  | NF |  |
| *ngo1252* | Q5F7C6 | membrane protein | 1.99 |  | COG0697 | E, G |
| *ngo1259* | Q5F7B9 | DNA topoisomerase IV subunit A | 1.50 |  | COG0188 | L |
| *ngo1260* | Q5F7B8 | hypothetical protein | 1.54 |  | COG2204 | T |
| *ngo1276* | Q5F7A4 | nitrite reductase, AniA | 1.52 | 2.36 | COG2132 | C |
| *ngo1288* | Q5F795 | membrane protein | 2.96 |  | NF |  |
| *ngo1290* | Q5F793 | alanine glycine permease | 2.33 |  | COG1115 | E |
| *ngo1292* | Q5F791 | disulfide bond formation protein B | 1.67 |  | COG1495 | O |
| *ngo1319* | Q5F767 | paraquot-inducible protein A | 1.94 |  | COG2995 | S |
| *ngo1355* | Q5F733 | sodium dependent ion transport protein | 4.81 |  | COG0733 | R |
| *ngo1359* | Q5F730 | hypothetical protein | 2.20 |  | NF |  |
| *ngo1360* | Q5F729 | GntR family transcriptional regulator | 2.10 |  | COG2186 | K |
| *ngo1395* | Q5F6Z4 | peptidase | 2.85 |  | COG3271 | R |
| *ngo1398* | Q5F6Z1 | membrane protein | 2.97 |  | COG1277 | R |
| *ngo1405* | Q5F6Y7 | hypothetical protein | 1.67 |  | NF |  |
| *ngo1407* | Q5F6Y5 | AsnC family transcriptional regulator | 1.55 |  | COG1522 | K |
| *ngo1455* | Q5F6U1 | membrane protein | 4.44 | 2.73 | COG1914 | P |
| *ngo1467* | Q5F6T0 | chloride channel protein | 1.75 |  | COG0038 | P |
| *ngo1497* | Q5F6Q2 | hypothetical protein | 3.39 |  | NF |  |
| *ngo1499* | Q5F6Q0 | FIC family protein | 1.80 |  | COG3177 | S |
| *ngo1513* | Q5F6N6 | Outer membrane opacity protein D, opaD | 2.62 | 2.20 | COG3637 | M |
| *ngo1528* | Q5F6M3 | cell division protein FtsZ | 1.72 |  | COG0206 | D |
| *ngo1544* | Q5F6K7 | mraW-Ribosomal RNA small subunit methyltransferase H | 1.58 |  | COG0275 | J |
| *ngo1558* | Q5F6J6 | hypothetical protein | 2.36 |  | NF |  |
| *ngo1581* | Q5F6H7 | phosphate permease | 1.55 |  | COG0306 | P |
| *ngo1603* | Q5F6F6 | mtgA-Monofunctional biosynthetic peptidoglycan transglycosylase | 1.69 |  | COG0744 | M |
| *ngo1615* | Q5F6E4 | hypothetical protein | 2.14 |  | COG0286 | V |
| *ngo1681* | Q5F681 | hypothetical protein | 2.84 |  | NF |  |
| *ngo1694* | Q5F669 | dihydrofolate reductase | 1.83 |  | COG0262 | H |
| *ngo1699* | Q5F667 | ABC transporter permease | 1.97 |  | COG4137 | O |
| *ngo1726* | Q5F640 | competence protein ComF family protein | 1.55 |  | COG1040 | R |
| *ngo1728* | Q5F638 | hypothetical protein | 2.94 | 6.07 | COG0797 | M |
| *ngo1729* | Q5F637 | hypothetical protein | 1.81 |  | COG3212 | S |
| *ngo1737* | Q5F629 | NADH-quinone oxidoreductase subunit N | 1.77 |  | COG1007 | C |
| *ngo1760* | Q5F606 | hypothetical protein | 4.26 |  | COG4304 | S |
| *ngo1768* | Q5F600 | membrane protein | 1.57 |  | COG1971 | S |
| *ngo1769* | Q5F5Z9 | cytochrome-c peroxidase | 2.57 |  | COG1858 | P |
| *ngo1792* | Q5F5X6 | membrane protein | 1.87 |  | NF |  |
| *ngo18781* | Q5F5N8 | hypothetical protein | 3.30 |  | NF |  |
| *ngo1888* | Q5F5N2 | hypothetical protein | 1.72 |  | NF |  |
| *ngo1929* | Q5F5J5 | hypothetical protein | 4.28 |  | NF |  |
| *ngo1930* | Q5F5J4 | DNA mismatch repair protein MutS | 2.50 | 2.05 | COG0249 | L |
| *ngo1932* | Q5F5J2 | hypothetical protein | 1.54 |  | COG1720 | S |
| *ngo2038* | Q5F591 | phosphoenolpyruvate-protein phosphotransferase | 1.56 |  | COG1080 | G |
| *ngo2043* | Q5F586 | hypothetical protein | 3.69 |  | COG1052 | H, C, R |
| *ngo2090* | Q5F546 | iron ABC transporter permease | 2.14 | 2.39 | COG4605 | P |
| *ngo2096* | Q5F540 | transporter | 1.63 |  | COG0733 | R |
| *ngo2121* | Q5F518 | lipoprotein VacJ | 2.67 | 4.31 | COG2853 | M |
| *ngo2122* | Q5F517 | hypothetical protein | 1.80 |  | NF |  |
| *ngo2123* | Q5F516 | thioesterase | 1.75 |  | COG0824 | S |
| *ngo2124* | Q5F515 | thioredoxin | 1.67 |  | COG0526 | O |
| *ngo2132* | Q5F508 | hypothetical protein | 1.57 |  | NF |  |
| *ngo2136* | Q5F504 | hypothetical protein | 4.24 |  | NF |  |
| *ngo2168* | Q5F4X5 | 3-oxoacyl-ACP synthase | 1.57 |  | COG0332 | I |
| *ngo2178* | Q5F4W6 | membrane protein insertase | 1.64 |  | COG0706 | U |
| *ngo2181* | Q5F4W3 | rnpA-Ribonuclease P protein component | 1.97 |  | COG0594 | J |
